# Nutrient deficiency effects on root architecture and root-to-shoot ratio in arable crops

**DOI:** 10.3389/fpls.2022.1067498

**Published:** 2023-01-04

**Authors:** Gina Lopez, Seyed Hamid Ahmadi, Wulf Amelung, Miriam Athmann, Frank Ewert, Thomas Gaiser, Martina I. Gocke, Timo Kautz, Johannes Postma, Shimon Rachmilevitch, Gabriel Schaaf, Andrea Schnepf, Alixandrine Stoschus, Michelle Watt, Peng Yu, Sabine Julia Seidel

**Affiliations:** ^1^ Crop Science, Institute of Crop Science and Resource Conservation, University of Bonn, Bonn, Germany; ^2^ Water Engineering Department, School of Agriculture, Shiraz University, Shiraz, Iran; ^3^ Drought Research Center, Shiraz University, Shiraz, Iran; ^4^ Soil Science, Institute of Crop Science and Resource Conservation, University of Bonn, Bonn, Germany; ^5^ Organic Farming and Cropping Systems, University of Kassel, Witzenhausen, Germany; ^6^ Directorate, Leibniz Centre for Agricultural Landscape Research (ZALF), Müncheberg, Germany; ^7^ Crop Science, Thaer-Institute of Agricultural and Horticultural Sciences, Humboldt-University of Berlin, Berlin, Germany; ^8^ Institute of Bio-Geosciences (IBG-2, Plant Sciences), Forschungszentrum Jülich GmbH, Jülich, Germany; ^9^ Blaustein Institutes for Desert Research, Ben Gurion University of the Negev, Beer Sheva, Israel; ^10^ Plant Nutrition Group, Institute of Crop Science and Resource Conservation, University of Bonn, Bonn, Germany; ^11^ Institute for Bio- and Geosciences (IBG-3, Agrosphere), Forschungszentrum Jülich GmbH, Jülich, Germany; ^12^ School of BioSciences, Faculty of Science, University of Melbourne, Melbourne, VIC, Australia; ^13^ Crop Functional Genomics, Institute of Crop Science and Resource Conservation, University of Bonn, Bonn, Germany; ^14^ Emmy Noether Group Root Functional Biology, Institute of Crop Science and Resource Conservation, University of Bonn, Bonn, Germany

**Keywords:** nutrient limitation, root plasticity, nitrogen, phosphorous, potassium, root morphology, fertilizer

## Abstract

Plant root traits play a crucial role in resource acquisition and crop performance when soil nutrient availability is low. However, the respective trait responses are complex, particularly at the field scale, and poorly understood due to difficulties in root phenotyping monitoring, inaccurate sampling, and environmental conditions. Here, we conducted a systematic review and meta-analysis of 50 field studies to identify the effects of nitrogen (N), phosphorous (P), or potassium (K) deficiencies on the root systems of common crops. Root length and biomass were generally reduced, while root length per shoot biomass was enhanced under N and P deficiency. Root length decreased by 9% under N deficiency and by 14% under P deficiency, while root biomass was reduced by 7% in N-deficient and by 25% in P-deficient soils. Root length per shoot biomass increased by 33% in N deficient and 51% in P deficient soils. The root-to-shoot ratio was often enhanced (44%) under N-poor conditions, but no consistent response of the root-to-shoot ratio to P-deficiency was found. Only a few K-deficiency studies suited our approach and, in those cases, no differences in morphological traits were reported. We encountered the following drawbacks when performing this analysis: limited number of root traits investigated at field scale, differences in the timing and severity of nutrient deficiencies, missing data (e.g., soil nutrient status and time of stress), and the impact of other conditions in the field. Nevertheless, our analysis indicates that, in general, nutrient deficiencies increased the root-length-to-shoot-biomass ratios of crops, with impacts decreasing in the order deficient P > deficient N > deficient K. Our review resolved inconsistencies that were often found in the individual field experiments, and led to a better understanding of the physiological mechanisms underlying root plasticity in fields with low nutrient availability.

## 1 Introduction

Sustainable intensification of agriculture is one promising way to meet the expected global increase in demand for food, fiber, fodder, and biofuel (Godfray and Garnett, 2014). However, edaphic stresses such as drought, soil nutrient availability, high acidity, and high salinity severely limit worldwide production. Managing nutrient deficiencies may be difficult, considering that the global efficiency of fertilizer application is frequently not more than 50% for nitrogen (N), less than 10% for phosphorus (P), and about 40% for potassium (K) (Fageria, 2012). Excessive fertilization may, in turn, promote groundwater pollution and gaseous N emissions. Hereby, the European Commission targets a 20% reduction in fertilizer quantities and a 50% reduction in nutrient losses by 2030 (European Commission, 2020).

Studies focusing on roots and on their role in nutrient acquisition are crucial to lay the basis of management strategies to increase crop production while improving resource use efficiency (Gregory et al., 2013). Root systems are strongly influenced by a wide range of abiotic factors such as gravity, soil compactness, soil water content, soil texture, aeration, nutrient availability, pH, and temperature (Yapa et al., 1988; Bengough et al., 2011; Kopke et al., 2015; Schneider et al., 2017; Hartmann et al., 2018; Correa et al., 2019; Hadir et al., 2021). Biotic factors (e.g., bacteria, fungi, nematodes, etc.) can also affect biogeochemical processes and affect the root morphology in the soil (Larsen et al., 2015). And vice versa, the root exudates stimulate microbial flora activity by fostering enzyme production. The microorganism decompose the soil organic matter, and consequently, the amounts of nutrients (N, P) increase, affecting the morphological traits in roots (Barrios-Masias et al., 2019)

Root systems can exhibit a high degree of plasticity in response to physical, chemical and biological changes in the environment (Lynch, 1995; Ostonen et al., 2007; Rich and Watt, 2013; Correa et al., 2019). For example, as reviewed by Correa et al. (2019), roots showed a retarded development as sign of apparent plasticity^1^, including changes in architecture, as a response to severe stress (e.g. soil compaction). These architectural changes may in turn enhance the tolerance to variations in the environmental conditions (adaptive plasticity). Drew et al. (1973) showed that plants grown on nutrient-rich soil patches increased number and length of fine lateral roots, thus positively affecting the overall specific root length (SRL).

Gruber et al. (2013) grew Arabidopsis plants on agar at four deficiency levels for 12 nutrients and quantified seven root traits. Total root length increased by 48% under moderate N deficiency and decreased under most severe N deficiency. Furthermore, since the root biomass decreased comparatively less than the shoot, the root-to-shoot ratio gradually increased with decreasing N supply. In addition, N deficiency stimulated the growth of a more exploratory root system with long lateral roots. Foehse and Jungk (1983) reported that some N deficiency level stimulates root hair formation of spinach, tomato, and rape in pot experiments. Additionally, plants grown at low N displayed longer root hairs than plants grown at higher N concentrations. Moreover, when oilseed rape was grown in a split-pot system, root hairs did not form when all root system grown in media with poor N supply, whereas root hairs were formed when at least part of the roots (10%) was grown in N-rich media.

Crops cope with P deficiency by increasing root development in the P-rich zone (commonly in the topsoil) (Lynch and Brown, 2001; Rogers and Benfey, 2015), releasing carboxylates that capture iron and aluminium from the respective phosphates, thus rendering P more soluble (Hodge et al., 2009), as well as directing arbuscular mycorrhizal uptake pathways (Smith et al., 2018). Total root length generally decreases with P deficiency (Gruber et al., 2013; Haling et al., 2018), and the growth of primary and lateral roots is restrained when roots reach a low-P zone (Desnos, 2008). However, roots can also develop a shallower, horizontal, and highly branched root system (Lynch and Brown, 2001; Gruber et al., 2013; Müller et al., 2015). For example, beans develop more horizontal root angles under P-limited soil, resulting in a more extensive root area in the topsoil, where P was more concentrated than in the subsoil (Bonser et al., 1996). Another well-known mechanism to enhance the P acquisition in P-limited conditions is the increase in length and number of root hairs (Schmidt, 2001; Lambers et al., 2006).

In contrast to the numerous studies investigating root responses to N and P deficiencies, research on the effects of K deficiency in roots is scarcer. Notably, a study with Arabidopsis showed a decrease in root biomass (about 60%) and primary root length at the lowest supplied K concentration, while root-to-shoot ratios remained stable across different levels of K deficiency (Gruber et al., 2013).

The current understanding of root plasticity has been mostly derived from seedlings and pot experiments conducted in controlled environments such as greenhouses or phytochambers (López-Bucio et al., 2003; Rich and Watt, 2013). However, the root growth behaviors in those conditions are frequently different than those observed under field conditions due to several abiotic and biotic factors, which are more variable and differ significantly from those in the greenhouse (Rich and Watt, 2013; Watt et al., 2013; Heinze et al., 2016; Schittko et al., 2016; Rich et al., 2020). Plants growing in fields are usually grown in crop stands, thus interacting and competing with each other, changing their environment and that of their neighboring plants (Cahill et al., 2010; Faget et al., 2013; Weidlich et al., 2018). Thus, pot studies generally do not have the physical, chemical and microbial composition of field soils. This difference alters the growth rate and rooting depth of plants as compared to field studies (Eno and Popenoe, 1964; De Deyn et al., 2004; Passioura, 2006; Ruzicka et al., 2010; Ruzicka et al., 2012; Poorter et al., 2016; Howard et al., 2017). For instance, Mokany and Ash (2008) found a poor correlation of root biomass and root-to-shoot ratio in pot experiments vs. field conditions. Moreover, the root responses to any stress differ in pots compared to field, as shown in cassava, where the root weight and width were statistically similar under drought and irrigated conditions at field scale but different in the pot experiments (Kengkanna et al., 2019). Another limitation in pot studies is that the container shape affects root morphological characteristics. Roots of plants cultivated in smooth-sided containers can grow deformed or limit their growth because they cannot spread horizontally, as they would do in an open field, therefore, they expand vertically, wrapping up at the bottom of the pot (Amoroso et al., 2010; Oburger and Schmidt, 2016). Besides, the container influences the humidity, and ventilation of soil (Poorter et al., 2012). Consequently, transferring observations on root morphology or plasticity from pot experiments to real field conditions is usually impossible.

To overcome these limitations, we performed a systemic review and meta-analysis to analyze whether and how N, P, and K deficiencies impact root morphological traits of common arable crops under field conditions. We were particularly interested in root length, root biomass, root diameter, root hair formation and root/shoot performance indices such as root-to-shoot ratio, root length per unit of shoot biomass and specific root length.

## 2 Materials and methods

We used a systematic review and meta-analysis approach to show the evidence of the effects of nutrient deficiencies on roots. The approach was as follows.

### 2.1 Data sources and search strategy

We used the electronic databases Web of Science, Google scholar, and Wiley online library to search for articles published in peer-reviewed journals without any restriction in the year of publishing. The exact combinations used for searching keywords was:

- Root + deficiencies + nutrients + field
- Root + nitrogen + field
- Root + nitrogen + site
- Root + phosphorus + field
- Root + phosphorus + site
- Root + potassium + field
- Root + potassium + site

In addition, secondary literature cited in selected papers was also looked up and included if relevant. In total, we considered 50 studies in which root growth of common field crops under field conditions was evaluated. All the key contents about the considered studies are summarized in the **Tables S1, S2, S3** of the **Supplementary Material**.

### 2.2 Selection criteria

The eligibility of the studies in this review was evaluated using the following criteria:

i. Investigation of roots, with observed data of at least one of the following traits: root growth, root length, root biomass, root-to-shoot ratio and/or root hair formation.
ii. Use of common agricultural crops.
iii. Reduction (or deficiency) of at least one of the three macro-nutrients N, P, or K, including a non-fertilized/insufficient control treatment.
iv. Experiments were conducted at a field-scale.

The exclusion criteria were:

i. Only qualitative data available.
ii. Forestry plants.
iii. Small-scale (e.g., pot or bucket experiments) or laboratory experiments (e.g., plants grown on agar).

### 2.3 Observed root traits

The following root traits were considered:

i. Root length and root length density (RLD)
ii. Root biomass
iii. Root mass density (RMD) or root weight density (RWD)
iv. Root length per shoot biomass
v. Root-to-shoot ratio
vi. Specific root length (SRL)
vii. Root diameter
viii. Root hair formation
ix. Speed of root growth
x. Root surface area

For definitions, please refer to the glossary provided by Freschet et al. (2021).

### 2.4 Data extraction

The extracted data for each study involved: i) name of the crop; ii) year of the study; iii) country of the experiment, iv) soil type; v) used method for root observation, vi) treatments; vii) effect on root morphology and distribution; and viii) effect on root length, root biomass, root diameter, shoot biomass and, root-to-shoot ratio, specific root length. Any other relevant information was also recorded and included in the text.

### 2.5 Estimation of the relative change of each trait due to nutrient deficiency

Besides an evaluation of the absolute trait values, the effect of the nutrient deficiency on each root trait was estimated using a relative change formula (Equation 1), where the value of the treatment without the specific nutrient was the comparison indicator.


(1) $$Relative\;Change=\frac{X0-X1}{X1}$$

Where X0 is the mean value of the trait (root length, biomass, etc.) without the nutrient application (e.g., 0 kg ha^-1^ of N) and X1 is the mean value of the trait with the nutrient addition (for example, application of 50 kg ha^-1^ of N). The relative change of root length, root biomass, root length per shoot biomass, root-to-shoot ratio, and diameter (if sufficient data was available) was calculated for each treatment and averaged for each study. Therefore, the mean of each study was considered as a single observation for the boxplots and the median estimation.

### 2.6 Statistical analysis

In order to compare the absolute values among the different studies, we normalized the absolute raw data with the following formula (Equation 2):


(2) $$Normalized\;value\;x'=\frac{\left(x-Xmin\right)}{\left(Xmax-Xmin\right)}$$

Where x is the absolute value of the root trait (root length, biomass, etc.) with respect to a specific nutrient availability level, Xmin is the lower bound in the values’ range (within the study and over all nutrient levels), and Xmax is the upper bound of the values’ range.

Then, we averaged the normalized data (grouped by deficiency or non-deficiency) to have two single observations per study (deficient and non-deficient). A normalized value close to 0 or 1 indicates that the value is similar to the study’s minimum or maximum values.

We then performed a t-test (t.test function of the stats R package) to compare the normalized data (one record per study if available) from deficient and non-deficient treatments and evaluate its statistical significance. The statistical analysis and all plots were created using the software R (version 4.0.2).

### 2.7 Considered studies

We found 32 studies that met the criteria of our search in the electronic databases and additional 18 publications cited within those studies. In total, 50 studies were analyzed in this work. We recognized that the keywords “field” and “site” were not often used in the titles or as keywords in our target studies, and thus additional papers were included through the references provided in the initially found manuscripts.

In the studies considered, the crops were grown in the USA, China, Australia, UK, Brazil, New Zealand, Iran, Costa Rica, Honduras, Canada, Mozambique, Colombia, Japan, Denmark, Germany and Belgium. Moreover, 29 out of 50 studies used fibrous root types (monocots) in their research, while the remaining 21 evaluated taproot root types (dicots). The studied crops are shown in **Table 1**.

**Table 1:** Number of studies per category at field-scale used for the systematic research.

Crop name (Latin name)		Number of studies per crop	Nutrient deficiency	Number of studies per nutrient deficiency
Barley	*(Hordeum vulgare)*	3	Nitrogen (N)	24
Common bean	*(Phaseolus vulgaris)*	7	Phosphorus (P)	19
Cotton	*(Gossypium)*	3	Potassium (K)	5
Maize	*(Zea mays)*	18	N and P	1
Millet	*(Pennisetum glaucum)*	1	N, P, and K	1
Oilseed rape	*(Brassica napus)*	2		
Potato	*(Solanum tuberosum)*	1		
Rice	*(Oryza sativa)*	1		
Sorghum	*(Sorghum)*	3		
Soybean	*(Glycine max)*	5		
Wheat	*(Triticum aestivum)*	6		
Sugarbeet	*(Beta vulgaris)*	1		
Sugarcane	*(Saccharum officinarum)*	1		
Buckwheat, castor, peanut, pigeon pea	(*Fagopyrum esculentum, Ricinus communis, Arachis hypogaea, Cajanus cajan)*	1		

## 3 Results

### 3.1 Relative change in root morphological traits under N, P, and K deficiency

**Figure 1:**
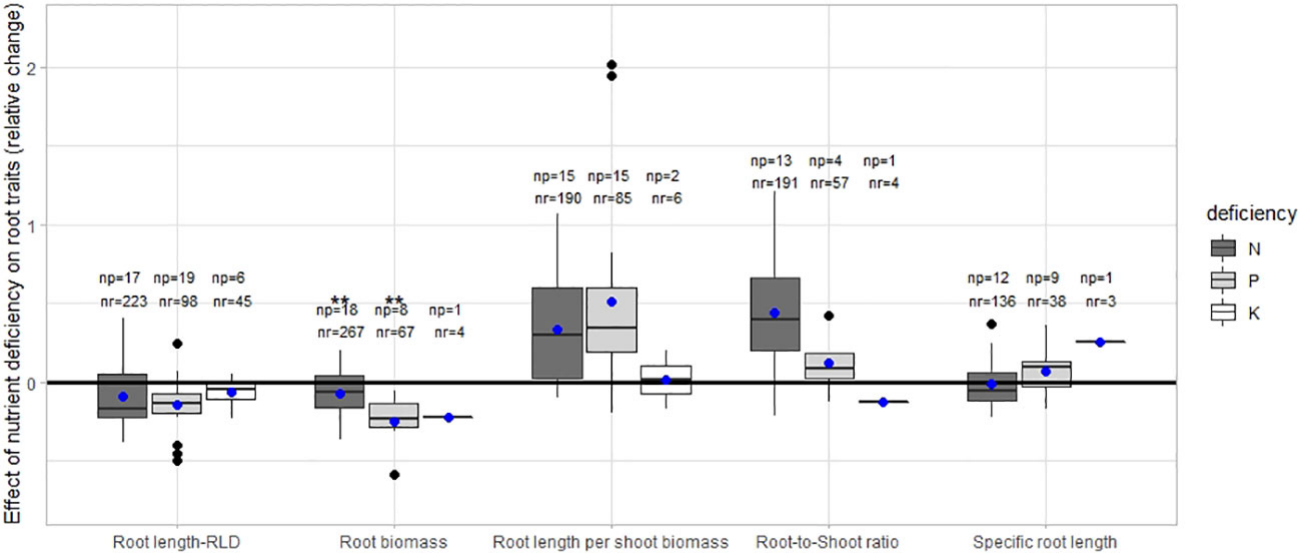
The relative change of the root traits under deficiency [(X0-X1)/X1] where X0 is the value in the treatment without any addition of the nutrient and X1 is the value of the treatment with the nutrient application. np stands for the number of publications/studies considered in the calculation, and nr for the total number of observations within these publications. The line within the boxes refers to the median. ** stands for significant differences at a 0.95 confidence level. Blue dots represent the mean.

Our meta-analysis revealed that root length and biomass, in most cases, decreased with increasing N, P, and K deficiency. Root length per shoot biomass and root-to-shoot ratio increased when plants were grown under N and P-deficient conditions. The specific root length was similar in nutrient-deficient and non-deficient treatments. The relative changes in root length, root biomass, root length per shoot biomass, root-to shoot ratio and specific root length under N, P and K deficiency are shown in **Figure 1**.

The magnitude (median) of the relative changes of the different root traits was similar among dicot and monocot plants under N and P deficiency (**Supplementary Figure S1**). The relative change of root-to-shoot ratio was greater under P deficiency than P-added in monocots plants.

### 3.2 Nitrogen

**Figure 2:**
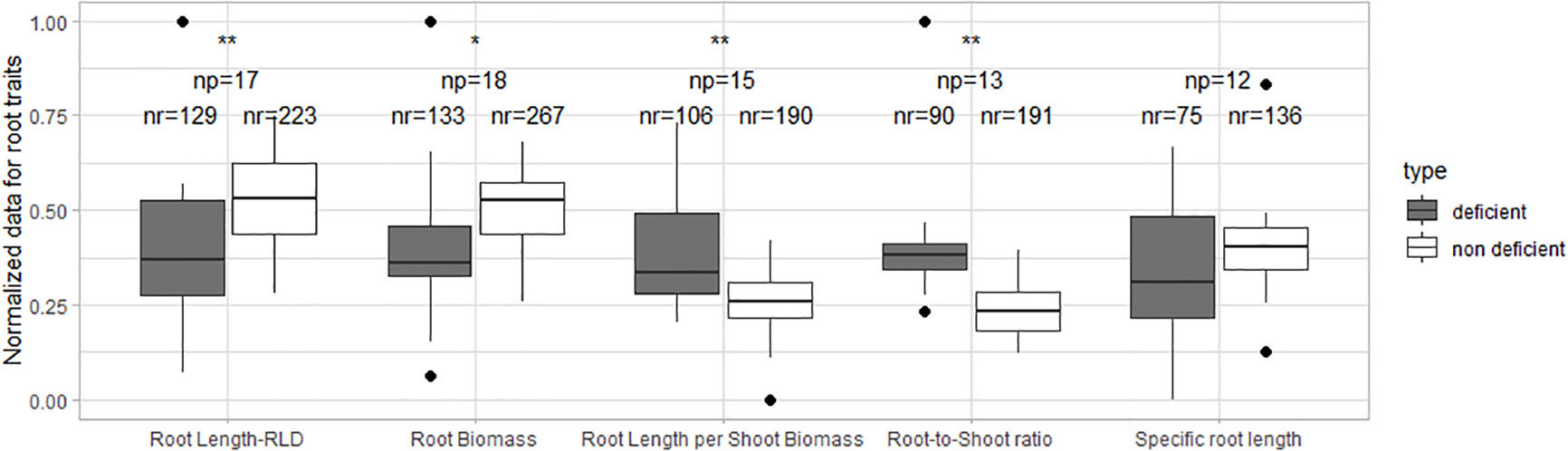
Boxplot of the normalized root data under N deficiency and N non-deficiency. A t-test was performed; * stands for significant differences at a 0.9 confidence level and ** at a 0.95 confidence level. np stands for the number of publications/studies considered in the calculation, and nr is the total number of observations within these publications (np).

The normalized root length, root biomass, root length per shoot biomass, and root-to-shoot ratio showed significant differences for N-deficient and non-deficient conditions (**Figure 2**). The normalized specific root length was similar in both treatments.

#### 3.2.1 Root length, root length density, and root surface area

**Table 2** shows an overview of the studies that report the effects of N deficiency on the total root length or RLD. It also shows the crop, soil type, factors investigated in each study, and the overall impact. Most of the observations revealed that absolute root length and RLD were lower under conditions of N deficiency than under sufficient N supply, particularly at N0^2^ (Barber and Mackay, 1986; Anderson, 1987; Anderson, 1988; Barraclough et al., 1989; Xue et al., 2014; Chen et al., 2020; Hadir et al., 2021; Mehrabi et al., 2021; Fang et al., 2022). This was observed for maize (Anderson, 1987; Anderson, 1988; Fang et al., 2022), winter wheat (Barraclough et al., 1989; Xue et al., 2014; Mehrabi et al., 2021), cotton and sugar beet (Chen et al., 2020; Hadir et al., 2021).

**Table 2:** Studies that report effects of N deficiency on total root length and/or root length density (TRL-RLD) at field scale.

Reference	Crop	Soil	Factors	TRL-RLD
(Chen et al., 2020)	cotton	loamy	LEV	DECREASE
(Anderson, 1987)	maize	silty loam	DEV YEAR	DECREASE
(Anderson, 1988)	maize	silty loam	DEV YEAR	DECREASE
(Fang et al., 2022)	maize	loamy	LEV YEAR DEV	DECREASE
(Hadir et al., 2021)	sugar beet	silty loam	DEV	DECREASE
(Barraclough et al., 1989)	winter wheat	silty clay loam	IRR DEV	DECREASE
(Mehrabi et al., 2021)	winter wheat		PLAN IRR LEV	DECREASE
(Xue et al., 2014)	winter wheat		DEV LEV	DECREASE
(Feng et al., 2016)	maize	loamy clay, clay loam, sandy loam	SOIL YEAR LEV	VARIABLE
(Mackay and Barber, 1986)	maize	silty loam	GEN DEV	VARIABLE
(Peng et al., 2012)	maize	silty loam	DEV LEV YEAR	VARIABLE
(Sharifi et al., 2005)	potato		GEN DEV	VARIABLE
(Nakamura et al., 2002)	sorghum		GEN DEV	VARIABLE
(Comfort et al., 1988)	spring wheat	silty loam, clay loam	GEN SITE LEV	VARIABLE
(Eghball and Maranville, 1993)	maize	silty clay loam	LEV	INCREASE
(NaNagara et al., 1976)	maize	silty loam	TILL DEV	INCREASE
(Thom and Watkin, 1978)	maize	sandy loam	DEV LEV	INCREASE

DECREASE (in red): diminished TRL-RLD, VARIABLE (in yellow): diverse, inconclusive or no effects on TRL-RLD, and INCREASE (in green): large TRL-RLD in case of deficient as compared to non-deficient conditions. Factors refer to the variables studied in each manuscript. LEV: several levels of N applied, DEV: several development stages investigated, YEAR: several years investigated, IRR: water treatments applied (such as irrigation and drought), PLAN: several planting methods tested, SOIL: several soil types tested, GEN: diverse genotype tested, SITE: different sites tested, TILL: several tillage practices tested. For more details refer to **SI Table 1**.

Some studies reported variable effects on root length and RLD depending on the other studied factors. In this line, Mackay and Barber (1986); Sharifi et al. (2005) and Nakamura et al. (2002) reported a genotype effect of N deficiency on maize, potato and sorghum root morphology. Feng et al. (2016) and Comfort et al. (1988) observed a weak parabolic relationship between N supply and root length (in maize and spring wheat). Peng et al. (2012) outlined that the effect of N deficiency on maize root length was related to the crop’s developmental stage. N deficiency (N0) stimulated root growth in early maize growth stages, and the total root length peaked before the tasseling, followed by an early decline compared with other treatments with increasing N supply in all three years studied.

In contrast, other studies found an increase in root length in N0 treatments. NaNagara et al. (1976); Thom and Watkin (1978), and Eghball and Maranville (1993) observed increased root lengths under zero N supply treatment compared with N-fertilized treatments of maize. Moreover, NaNagara et al. (1976) found that the effects of N fertilization on root length interacted with the tillage regime and development stage.

#### 3.2.2 Root biomass

**Table 3** summarizes the main effects of N deficiency on root biomass. Most of the observations show a decrease in the total root biomass in the N0 treatment, regardless the crop (Welbank and Williams, 1968; Myers, 1980; Barraclough et al., 1989; Nakamura et al., 2002; Sharifi et al., 2005; Otto et al., 2014; Xue et al., 2014; Schneider et al., 2017; Mehrabi et al., 2021; Fang et al., 2022), crop developmental stage (Myers, 1980; Barraclough et al., 1989; Nakamura et al., 2002; Sharifi et al., 2005; Xue et al., 2014; (Welbank and Williams, 1968), genotype (Schneider et al., 2017; Fang et al., 2022), or irrigation regimens (Mehrabi et al., 2021).

**Table 3:** Studies that report effects of N deficiency on root biomass (RBIO) at field scale.

Reference	Crop	Soil	Factors	RBIO
(Welbank and Williams, 1968)	barley		DEV LEV	DECREASE
(Fang et al., 2022)	maize	loamy	LEV YEAR DEV	DECREASE
(Schneider et al., 2017)	maize	silt loam, clay loam	SOIL	DECREASE
(Sharifi et al., 2005)	potato		GEN DEV	DECREASE
(Nakamura et al., 2002)	sorghum		GEN DEV	DECREASE
(Myers, 1980)	sorghum	clay loamy	GEN DEV	DECREASE
(Otto et al., 2014)	sugarcane	Typic Kandiudox, Rhodic Eutrudox	SOIL LEV DEV	DECREASE
(Barraclough et al., 1989)	winter wheat	silty clay loam	IRR DEV	DECREASE
(Mehrabi et al., 2021)	winter wheat		PLAN IRR LEV	DECREASE
(Xue et al., 2014)	winter wheat		DEV LE	DECREASE
(Chen et al., 2020)	cotton	loamy	LEV	VARIABLE
(Sainju et al., 2005)	cotton	sandy loam	TILL LEV	VARIABLE
(Anderson, 1987)	maize	silty loam	DEV YEAR	VARIABLE
(Anderson, 1988)	maize	silty loam	DEV YEAR	VARIABLE
(Feng et al., 2016)	maize	loamy clay, clay loam, sandy loam	SOIL YEAR LEV	VARIABLE
(Thom and Watkin, 1978)	maize	sandy loam	DEV LEV	VARIABLE
(Hadir et al., 2021)	sugar beet	silty loam	DEV	VARIABLE
(Wang et al., 2005)	winter wheat	clay loamy	IRR LEV YEAR	VARIABLE
(Eghball and Maranville, 1993)	maize	silty clay loam	LEV	INCREASE

DECREASE (in red): diminished RBIO, VARIABLE (in yellow): diverse, inconclusive or no effects on RBIO, and INCREASE (in green):higher RBIO in case of deficient as compared to non-deficient conditions. Factors refer to the variables studied in each manuscript. LEV: several levels of N applied, DEV: several development stages investigated, YEAR: several years investigated, IRR: water treatments applied (such as irrigation and drought), PLAN: several planting methods tested, SOIL: several soil types tested, GEN: diverse genotype tested, SITE: different sites tested, TILL: several tillage practices tested. For more details refer to **SI Table 1**.

Some studies found variable effects on root biomass depending on the other studied factors. Anderson (1987, 1988) reported that tillage treatments and year of cultivation affected maize root morphology differently under N deficiency. In the 3-year field experiment in three different soils types (loamy clay, clay loam, and sandy loam) conducted by Feng et al. (2016), less maize root biomass was found in N0 treatment, except in the loamy clay in one out of the three years. In another maize study, at early and grain-filling stages, plants grown under N0 conditions presented higher root dry weight than those submitted to N168 and N672 treatments (Thom and Watkin, 1978). In winter wheat, Wang et al. (2014) found that the effect of N on root weight density depended on soil water conditions. Root biomass under N deficiency reacted differently depending on the development stages (Hadir et al., 2021), level of deficiency (Chen et al., 2020), and tillage (Sainju et al., 2005).

In contrast, only one study (Eghball and Maranville, 1993) reported increased maize root biomass under N deficiency and no interactions with the maize genotype. Dry maize root weight at N0 was higher than at N60, N120, and N180.

#### 3.2.3 Root-to-shoot ratio

**Table 4** describes the effects of N deficiency on two ratios: root-to-shoot and root length per shoot biomass, including the soil type and variables investigated in each study. Most of the studies reported an increase in the root-to-shoot ratio upon N deprivation (Welbank and Williams, 1968; Welbank and Williams, 1968; Myers, 1980; Anderson, 1988; Eghball and Maranville, 1993; Sharifi et al., 2005; Wang et al., 2005; Farrior et al., 2013; Xue et al., 2014; Hadir et al., 2020), indicating a greater investment of assimilates into the belowground crop parts under low N conditions (**Figure 2**).

**Table 4:** Studies that report effects of N deficiency on the root-to-shoot ratio (R_S) and root length per shoot biomass (LENG_SHOOT) at the field scale.

Reference	Crop	Soil	Factors	R_S	LENG_SHOOT
(Sharifi et al., 2005)	potato		GEN DEV	INCREASE	INCREASE
(Xue et al., 2014)	winter wheat		DEV LEV	INCREASE	INCREASE
(Eghball and Maranville, 1993)	maize	silty clay loam	LEV	INCREASE	VARIABLE
(Hadir et al., 2021)	sugar beet	silty loam	DEV	INCREASE	DECREASE
(Welbank and Williams, 1968)	barley		DEV LEV	INCREASE	
(Anderson, 1988)	maize	silty loam	DEV YEAR	INCREASE	
(Myers, 1980)	sorghum	clay loamy	GEN DEV	INCREASE	
(Wang et al., 2005)	winter wheat	clay loamy	IRR LEV YEAR	INCREASE	
(Feng et al., 2016)	maize	loamy clay, clay loam, sandy loam	SOIL YEAR LEV	VARIABLE	VARIABLE
(Otto et al., 2014)	sugarcane	Typic Kandiudox, Rhodic Eutrudox	SOIL LEV DEV	VARIABLE	
(Fang et al., 2022)	maize	loamy	LEV YEAR DEV	DECREASE	DECREASE
(Louvieaux et al., 2018)	oilseed rape		DEV		INCREASE
(Nakamura et al., 2002)	sorghum		GEN DEV		INCREASE
(Comfort et al., 1988)	spring wheat	silty loam, clay loam	GEN SITE LEV		INCREASE
(Peng et al., 2012)	maize	silty loam	DEV LEV YEAR		VARIABLE

DECREASE (in red): diminished R_S, LENG_SHOOT, VARIABLE (in yellow): diverse, inconclusive or no effects on R_S, LENG_SHOOT, and INCREASE (in green): higher R_S, LENG_SHOOT in case of deficient as compared to non-deficient conditions. Factors refer to the variables studied in each manuscript. LEV: several levels of N applied, DEV: several development stages investigated, YEAR: several years investigated, IRR: water treatments applied (such as irrigation and drought), PLAN: several planting methods tested, SOIL: several soil types tested, GEN: diverse genotype tested, SITE: different sites tested, TILL: several tillage practices tested. For more details refer to **SI Table 1**.

Two studies reported variable effects on the root-to-shoot ratio depending on the other studied factors. Feng et al. (2016) reported that the root-to-shoot ratio of maize at silking was higher in N0, except in the loamy clay soil in one out of the three years of the study. In sugarcane, N deficiency led to a decrease in root-to-shoot ratio at the beginning of the production cycle at one out of two experimental sites. In later growth stages, the root-to-shoot ratio was similar between the treatments (Otto et al., 2014).

#### 3.2.4 Root diameter, root diameter distribution, and specific root length

All the studies that investigated the effect of N deficiency on root diameter and specific root length are listed in **Table 5**. Only a few studies reported observations of root radius, root diameter, root diameter distribution, or specific root length, and a predominant effect of N treatments on these traits cannot be identified. An increase in maize average root diameter in N0 as compared to N180 was observed in a long-term experiment (Anderson, 1987). In contrast, Sharifi et al. (2005) reported no effect of low N conditions on root diameter for potato. Otherwise, a decrease in average root diameter at N0 was reported for maize (Eghball and Maranville, 1993) and sugar beet experiment (Hadir et al., 2021).

**Table 5:** Studies that report effects of N deficiency on the root diameter (DIA) and specific root length (SRL) at the field scale.

Reference	Crop	Soil	Factors	DIA	SRL
(Anderson, 1987)	maize	silty loam	DEV YEAR	INCREASE	INCREASE
(Sharifi et al., 2005)	potato		GEN DE	VARIABLE	
(Eghball and Maranville, 1993)	maize	silty clay loam	LEV	DECREASE	
(Hadir et al., 2021)	sugar beet	silty loam	DEV	DECREASE	
(Fang et al., 2022)	maize	loamy	LEV YEAR DEV		INCREASE
(Nakamura et al., 2002)	sorghum		GEN DEV		INCREASE
(Anderson, 1988)	maize	silty loam	DEV YEAR		VARIABLE
(Mehrabi et al., 2021)	winter wheat		PLAN IRR LEV		DECREASE

DECREASE (in red): diminished DIA/SRL, VARIABLE (in yellow): diverse, inconclusive or no effects on DIA/SRL, and INCREASE (in green): higher DIA/SRL in case of deficient as compared to non-deficient conditions. Factors refer to the variables studied in each manuscript. LEV: several levels of N applied, DEV: several development stages investigated, YEAR: several years investigated, IRR: water treatments applied (such as irrigation and drought), PLAN: several planting methods tested, SOIL: several soil types tested, GEN: diverse genotype tested, SITE: different sites tested, TILL: several tillage practices tested. For more details refer to **SI Table 1**.

Higher values of specific root length at N0 were found in maize (Anderson, 1987; Fang et al., 2022) and sorghum (Nakamura et al., 2002). In contrast, Mehrabi et al. (2021) reported a smaller SRL when N was not applied.

#### 3.2.5 Other effects on root morphology

Barber and Mackay (1986) conducted a field experiment with two different maize genotypes in two different soils. The percentage of roots with root hairs was not affected by the amounts of applied N (N0 and N227), but N0 led to a decrease in both root number and root hair length in all maize genotypes.

Schneider et al. (2021) found that maize lines with few-thick nodal roots had smaller total axial root lengths in N0, while lines with many-thin developed a greater total axial root length in N0. The phenotype of fewer, thicker nodal roots was associated with deeper root distribution and resulted in an increased shoot growth under N deficiency.

Maize showed a decrease in the speed of root growth rate (30-49% less) in the topsoil (0-25cm) but an increase (50-60% more) in the subsoil (26-80cm) in treatment N0 compared with N227 at the early growth stage (Barber and Mackay, 1986).

### 3.3 Phosphorus

A summary of the experimental setup and main effects of P deficiency in root morphology and topology is provided in **Table S2**.

**Figure 3:**
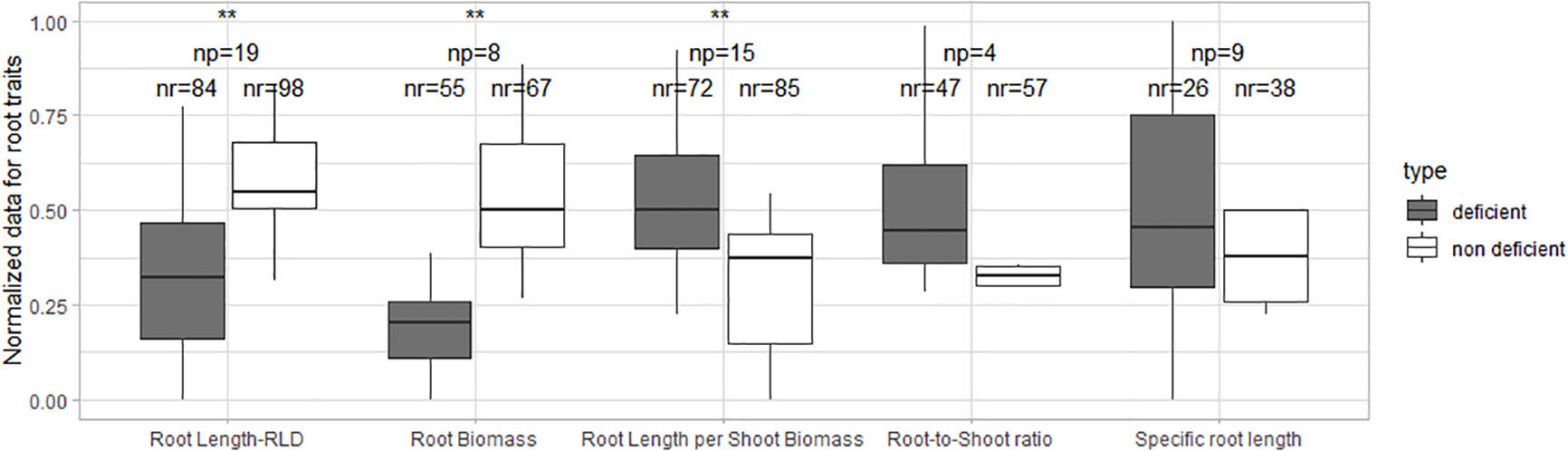
Boxplot of the normalized root data under P deficiency and P non-deficiency. A t-test was performed; ** stands for significant differences at a 0.95 confidence level. np stands for the number of publications/studies considered in the calculation, and nr is the total number of observations within these publications (np).

Normalized data of root length, root biomass, and root length per shoot biomass differ significantly between P-deficient and non-deficient treatments (**Figure 3**). The differences in root-to-shoot ratio and specific root length were non-significant.

#### 3.3.1 Root length and root length density

**Table 6** summarizes the studies that report the effects of P deficiency on the total root length or RLD, describing the crop, soil type, factors investigated in each study, and the overall impact. Most studies reported a decrease in root length or root length density under P deficiency (**Figure 3**). This was the case for maize (Sheng et al., 2012; Zhang et al., 2012; Deng et al., 2014), oilseed rape (Duan et al., 2020), sugar beet (Hadir et al., 2020), soybean (Otani and Ae, 1996; Ao et al., 2010), common beans (Ho et al., 2005; Ochoa et al., 2006; Miguel et al., 2015), wheat (Teng et al., 2013), as well as for buckwheat, castor, peanut and sorghum (Otani and Ae, 1996). Although all these authors reported a decrease in root length under P deficiency conditions (P0), there are some particularities. For example, in two studies, several (six and eight) P levels were tested. In both cases, root length and/or RLD increased with P-fertilizer rate at first, peaked, and then either declined again in the case of wheat (Teng et al., 2013) or reached a plateau in the case of maize (Deng et al., 2014).

**Table 6:** Studies that report effects of P deficiency on total root length and/or root length density (TRL-RLD) and root biomass (RBIO) at field scale.

Reference	Crop	Soil	Factors	TRL-RLD	RBIO
(Ho et al., 2005)	common beans		GEN IRR	DECREASE	
(Miguel et al., 2015)	common beans	loamy	GEN	DECREASE	
(Ochoa et al., 2006)	common beans		GEN	DECREASE	DECREASE
(Deng et al., 2014)	maize	silty loam	LEV	DECREASE	DECREASE
(Sheng et al., 2012)	maize	clay loamy	LEV	DECREASE	DECREASE
(Zhang et al., 2012)	maize	loamy and silt	DEV	DECREASE	DECREASE
(Duan et al., 2020)	oilseed rape	Alfisol	DEV GEN	DECREASE	DECREASE
(Ao et al., 2010)	soybean	Acidic red soil	GEN	DECREASE	
(Hadir et al., 2021)	sugar beet	silty loam	DEV	DECREASE	DECREASE
(Teng et al., 2013)	wheat	silty	LEV YEAR	DECREASE	DECREASE
(Henry et al., 2010b)	common beans		GEN IRR	VARIABLE	
(Henry et al., 2010a)	common beans		GEN	VARIABLE	
(Miguel et al., 2013)	common beans	loamy	GEN	VARIABLE	
(Strock et al., 2018)	common beans	silty loam	GEN	VARIABLE	
(Li et al., 2017)	maize	clay loamy	LEV	VARIABLE	DECREASE
(Otani and Ae, 1996)	others		CROP	VARIABLE	
(Jing et al., 2004)	soybean	Acidic red soil	GEN	VARIABLE	
(Steingrobe et al., 2001)	winter barley	loamy	DEV	VARIABLE	
(Gutierrez-Boem and Thomas, 1998)	soybean	silty	IRR LEV	INCREASE	

DECREASE (in red): diminished TRL-RLD/RBIO, VARIABLE (in yellow): diverse, inconclusive or no effects on TRL-RLD/RBIO, and INCREASE (in green):a large TRL-RLD/RBIO in case of deficient as compared to non-deficient conditions. Factors refer to the variables studied in each manuscript. LEV: several levels of N applied, DEV: several development stages investigated, YEAR: several years investigated, IRR: water treatments applied (such as irrigation and drought), PLAN: several planting methods tested, SOIL: several soil types tested, GEN: diverse genotype tested, SITE: different sites tested, TILL: several tillage practices tested, CROP: several crops tested. For more details refer to **SI Table 2**.

Some studies described that root length was not affected only by P deficiency but also by interactions with other factors. For instance, a genotype effect was found for common beans (Henry et al., 2010a; Henry et al., 2010b; Miguel et al., 2013; Strock et al., 2018) and soybean (Jing et al., 2004). In maize, the level of P deficiency caused diverse effects in RLD (Li et al., 2017). Moreover, the root length of winter barley reacted differently along the development stages at P0 (Steingrobe et al., 2001).

On the contrary, only one study in soybean reported an increase in the root length density in P0, particularly at the topsoil. Nevertheless, no differences were observed in the subsoil (Gutierrez-Boem and Thomas, 1998).

#### 3.3.2 Root biomass

In most studies, deficiency of P supply decreased absolute root biomass (**Figure 1**), as found in maize (Sheng et al., 2012; Zhang et al., 2012; Deng et al., 2014; Li et al., 2017), oilseed rape (Duan et al., 2020), sugar beet (Hadir et al., 2021), wheat (Teng et al., 2013) and common bean (Ochoa et al., 2006). However, a P oversupply could also decrease the root biomass. For instance, in the studies with maize (Deng et al., 2014; Li et al., 2017) and winter wheat (Teng et al., 2013) where several P levels were tested, root dry weight initially increased with increasing soil P supply, reaching its peak and then gradually declined in case of oversupply of P.

#### 3.3.3 Root-to-shoot ratio

Few studies reported the effect of P deficiency on the root-to-shoot ratio (**Table 7**); therefore, it is not possible to conclude about the effect of P deficiency on this trait. An increase in root-to-shoot in P0 compared to high P treatments was found for wheat (Teng et al., 2013) and maize (Deng et al., 2014). In oilseed rape, the root-to-shoot ratio was higher or smaller depending on the genotype under P stress (Duan et al., 2020). Only one study (in sugar beet) reported a decrease in the root-to-shoot ratio under P deficiency (Hadir et al., 2021).

**Table 7:** Studies that report effects of P deficiency on the root-to-shoot ratio (R_S), root length per shoot biomass (LEGN_SHOOT), root diameter (DIA) and specific root length (SRL) at the field scale.

Reference	Crop	Soil	Factors	R_S	LENG_SHOOT	DIA	SRL
(Deng et al., 2014)	maize	silty loam	LEV	INCREASE	INCREASE		INCREASE
(Teng et al., 2013)	wheat	silty	LEV YEAR	INCREASE	VARIABLE		
(Duan et al., 2020)	oilseed rape	Alfisol	DEV GEN	VARIABLE	VARIABLE		INCREASE
(Hadir et al., 2021)	sugar beet	silty loam	DEV	DECREASE	DECREASE		DECREASE
(Zhang et al., 2012)	maize	loamy and silt	DEV		INCREASE	DECREASE	VARIABLE
(Ho et al., 2005)	common beans		GEN IRR		INCREASE		
(Miguel et al., 2015)	common beans	loamy	GEN		INCREASE		
(Henry et al., 2010b)	common beans		GEN IRR		INCREASE		
(Gutierrez-Boem and Thomas, 1998)	soybean	silty	IRR LEV		INCREASE		
(Sheng et al., 2012)	maize	clay loamy	LEV		VARIABLE	DECREASE	VARIABLE
(Henry et al., 2010a)	common beans		GEN		VARIABLE		
(Jing et al., 2004)	soybean	Acidic red soil	GEN		VARIABLE		
(Steingrobe et al., 2001)	winter barley	loamy	DEV		VARIABLE		
(Ao et al., 2010)	soybean	Acidic red soil	GEN		DECREASE		VARIABLE
(Li et al., 2017)	maize	clay loamy	LEV			VARIABLE	VARIABLE
(Ochoa et al., 2006)	common beans		GEN				INCREASE

DECREASE (in red): diminished effect, VARIABLE (in yellow): diverse, inconclusive or no effects, and INCREASE (in green): higher effect in case of deficient as compared to non-deficient conditions. Factors refer to the variables studied in each manuscript. LEV: several levels of N applied, DEV: several development stages investigated, YEAR: several years investigated, IRR: water treatments applied (such as irrigation and drought), PLAN: several planting methods tested, SOIL: several soil types tested, GEN: diverse genotype tested, SITE: different sites tested, TILL: several tillage practices tested, CROP: several crops tested. For more details refer to **SI Table 2**.

#### 3.3.4 Root diameter, root diameter distribution, and specific root length

Few studies reported the effect of P deficiency on root diameter distribution (**Table 7**). In maize, a decrease in root diameter was observed in P0 compared to the plants that received P fertilizer at the vegetative stage, jointing, and silking (Sheng et al., 2012; Zhang et al., 2012). On the other hand, Li et al. (2017) found no differences in the maize mean root diameter among the tested P treatments.

A P deficiency led to a higher specific root length in oilseed rape (Duan et al., 2020), in maize (Deng et al., 2014), and in common bean (Ochoa et al., 2006) (**Table 7**). However, in maize, some specificities were found; for instance, Li et al. (2017) observed a higher SRL in P0 compared with P35 but lower compared with P18. On the contrary, Sheng et al. (2012) reported lower maize SRL in P0 compared with P18 but higher in P35, and Zhang et al. (2012) observed a higher SRL in P0, except before flowering. In soybean, the SRL increased in one genotype under low P and decreased in the other (Ao et al., 2010). Furthermore, in sugar beet, the SRL was smaller in the P0 treatment in a long-term field experiment (Hadir et al., 2021).

#### 3.3.5 Other effects on root morphology

Zhu et al. (2010) found that genotypes with long root hairs under low P availability had significantly higher plant growth, P uptake, specific P absorption rates, and lower metabolic cost-benefit ratios than short-haired genotypes. In this work, root hairs were also longer in the low P treatment.

An increment in relative basal root fraction in common beans at low P was observed by Ho et al. (2005).

Steingrobe et al. (2001) grew winter barley in plots that had received 0 and 44 kg P ha^-1^ over 14 years. The authors observed a faster root production (root dry weight increment per shoot increment) of winter barley in treatments with P0 compared with P44 in all the vegetative stages.

### 3.4 Potassium

Only six studies that investigated the effect of K deficiency on root growth were identified. A summary of their setup and major findings are described in **Supplementary Table 3**.

**Figure 4:**
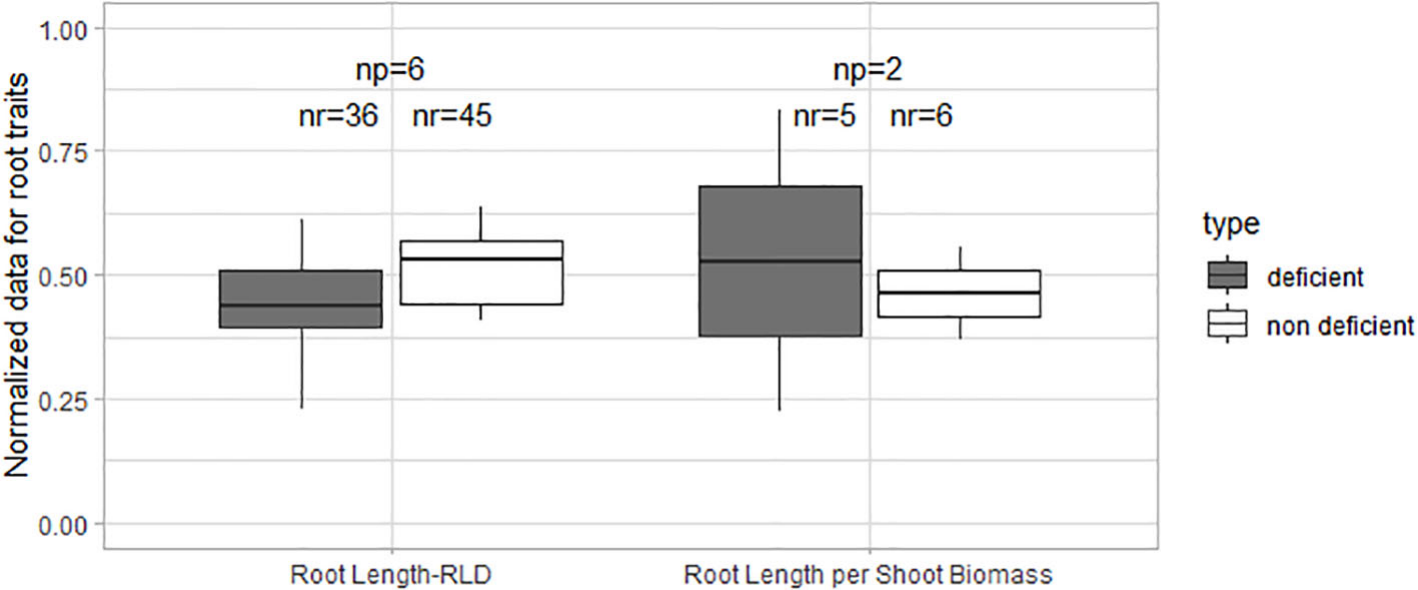
Boxplot of the normalized root data under K deficiency and K non-deficiency. A t-test was performed; no significant differences were found. np stands for the number of publications/studies considered in the calculation and nr the total number of observations within these publications (np).

Normalized data of root length and root length per shoot biomass did not show significant differences in these traits between K-deficient and non-deficient treatments (**Figure 4**). Studies of K deficiency did not provide enough data on root biomass, root-to-shoot ratio, and specific root length to perform statistical analysis. However, some effects are described in the sections below.

#### 3.4.1 Root length and root length density

Most studies reported smaller (but not significant) root lengths or RLD under low K conditions (**Table 8**). For example, in cotton (Mullins et al., 1994), in sugar beet (Hadir et al., 2020), millet (Valadabadi and Farahani, 2009; Zhao et al., 2016), and maize (Zhao et al., 2016).

**Table 8:** Studies that report effects of K deficiency on total root length and/or root length density (TRL-RLD) at field scale.

Reference	Crop	Soil	Factors	TRL-RLD
(Mullins et al., 1994)	cotton	sandy loam	YEAR	DECREASE
(Zhao et al., 2016)	maize	sandy	GEN	DECREASE
(Hadir et al., 2021)	sugar beet	silty loam	DEV	DECREASE
(Andersen et al., 1992)	barley	sandy	YEAR DEV	VARIABLE
(Valadabadi and Farahani, 2009)	maize, sorghum and millet	sandy loam	IRR	VARIABLE
(Fernández et al., 2009)	soybean	silty loam	YEAR LEV DEV	VARIABLE

Some studies found variable effects on root length and RLD depending on the other studied factors. In barley, Andersen et al. (1992) did not detect significant differences between the medium and high K treatments (K50 and K200) in one year, while in the other year, the root density in the subsoil layers significantly increased by application of high K amounts (K200). In soybean, Fernández et al. (2009) found longer root lengths under low K conditions compared with medium and high K treatments in one of the two years of the experiment, andthe root length was smaller in low K treatments in the second year.

#### 3.4.2 Root biomass, root-to-shoot ratio, and root diameter

K0 led to a decrease in sugar beet root biomass and root-to-shoot ratio in a long-term field experiment (Hadir et al., 2021).

In soybean, a decrease in the average root diameter in low K conditions was observed throughout the growing period (Fernández et al., 2009) and at the seedling and shooting stages (Zhao et al., 2016). The average root diameter was similar in booting and tasseling in the study of Zhao et al. (2016).

### 3.5 Summary of the effects of nutrient deficiencies on root morphological traits

**Figure 5:**
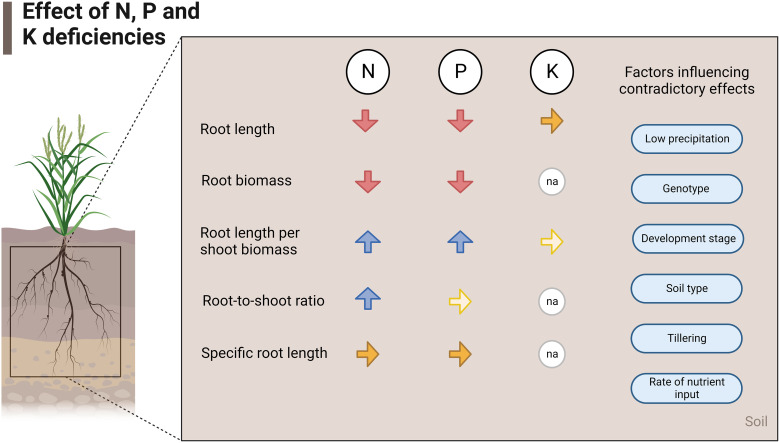
Effects of N, P, and K deficiencies at field scale. The red arrows show a decrease, the blue arrows show an increase and the yellow arrows show similarity in that trait in case of deficiency of the respective nutrient. Lighter-colored arrows stand for few studies found investigating that specific parameter (2-4 studies), and na stands for not applicable.

**Figure 5** summarizes the effects of nutrient deficiencies on five root traits evaluated in this study based on the relative change and normalized values of root traits. Also, the factors that influence contradictory effects in field experiments are listed (**Figure 5**).

N and P deficiencies in field crop production frequently lead to the reduction of absolute root length, RLD, and absolute root biomass but to an increase of root length per shoot biomass (**Figures 1**, **5**). Moreover, the root-to-shoot ratio increased under low N conditions. Few studies investigated the effects of low P on root-to-shoot, and no statistical differences were found in the normalized data between P deficient and non-deficient treatments. Specific root length was also statistically similar under N and P-deficiency and non-deficiency treatments. The lack of studies on the effects of K deficiency on root morphology limited the assessment of all the traits covered in this review. However, the available data showed that root length and root length per shoot biomass were similar in control and K-sufficient treatments.

## 4 Discussion

The spatial-temporal fluctuations and occurrences of nutrients in the soil are monitored by sensory mechanisms at root tips. This information triggers chemical signals which may shape root growth (Asim et al., 2020). The decrease in root length and root biomass upon N and P deficiency (see also **Figures 1**-**3**) seems to be a general property of root morphological plasticity. The low N and P availability negatively affects the above-ground part of the plant, including the leaf area and the photosynthetic capacity per unit of leaf area, consequently leading to a decrease in carbohydrates to be invested in root growth (Postma et al., 2014). Initially, a reduction in photosynthesis might be offset by an increase in the allocation of photosynthates to roots in order to maintain root growth. However, this resource relocation leads to a more pronounced shoot growth reduction, possibly limiting light capture and photosynthesis even more. Eventually, the smaller plants cannot sustain proper root and shoot growth, and absolute root length and biomass decrease.

Noteworthy, the above-mentioned general trend has exceptions. Some studies reported plants with longer roots in low nutrient conditions. In principle, the increase in root length could be a temporary effect in the early development stages (Peng et al., 2012; Xue et al., 2014). On the other hand, it could be that early investment in root growth under low nutrient conditions represents an advantageous strategy to cope with nutrient deprivation, e.g., as a tool to forage into the subsoil (Jia et al., 2022). Several reports indicate that the contribution of subsoil nutrients to overall uptake can be quite variable (Kautz et al., 2013) and also depend on other factors. Those include penetration resistance (Schneider et al., 2017), water distribution in the soil, as well as the availability of other nutrients, e.g., N abundancy when P is deficient (Bauke et al., 2017)

An apparent effect of N and P deficiencies on root morphology is the higher ratio between root length and shoot biomass. This may be explained by the enormous negative impact of N and P starvation on above-ground biomass, estimated at about 34% of shoot biomass decrease when N or P is deficient. Indeed, the root length also decreases due to the N and P deficiency, but not as much as the shoot biomass. Our review shows a decrease in root length of about 20% for N deficiency and 15% for P deficiency (**Figure 1**), which is lower than the decrease in above-ground biomass.

Most of the root-to-shoot ratios (**Figure 2**) increased under N deficiency. It is well known that the root-to-shoot ratio increases under N deficiency due to the concept of functional equilibrium. Competition for carbohydrates and nitrogenous compounds regulates root-to-shoot ratios. For example, when plants are changed from a non-N environment to an N environment with sufficient N supply, the shoot increases its growth in the short term, switching to a lower root-to-shoot ratio and delaying the root growth (Ågren and Ingestad, 2006). On the other hand, when the plant is transferred from a high N level to a zero N level, a non-equilibrium scenario appears; in the beginning, the ratio does not change much as long as free nitrate is available in the tissue, but when the internal nitrate content is depleted, the redistribution of organic-N determines the growth rate (Brouwer, 1983). In that scenario, root growth increases gradually more than shoot growth (Brouwer, 1983). In the end, shoot growth decreases when all the compounds are in N equilibrium. When the plants grow in a prolonged N-deficiency environment, the response to a renewed supply of N decreases (Brouwer, 1983).

Greenhouse (Horst et al., 1996; Shen et al., 2018) and lab studies (Rychter and Randall, 1994; Mollier and Pellerin, 1999; Ciereszko et al., 2011) have shown that the root-to-shoot ratio increases in low P conditions. However, our study could not confirm this finding, possibly due to the sample size (only three studies) which was too small to compare the effect between different conditions.

Specific root length was not affected by N or P deficiency consistently. For example, Ostonen et al. (2007) found a higher SRL in treatments with low nutrient levels. However, this finding was related only to the finest roots, and our review lacks the differentiation of root types. Poorter and Ryser (2015) have analyzed the response of specific leaf area (SLA) to light constraints and the specific root length (SRL) to nutrient availability constraints, as a similar response to constraints above and below ground crop parts, respectively. The changes in SRL were not as significant as SLA changes. However, by separating the root types by function (primary roots from lateral roots), the authors found that low nutrient levels positively affect the SRL of the lateral roots, which are supposedly most active in resource acquisition.

Due to a lack of data, our study can only conclude one consistent result with respect to the effect of K, which is the reduction in root length under K deficiency conditions. This observation can be explained, as in the case of N and P deficiency, with the lower availability of assimilates when K availability is reduced.

Interestingly, the root types monocot and dicot do not only share similar root morphology responses to N and P deficiency but also do so in similar magnitude (see **Supplementary Figure S1**), despite the differences in their root systems. However, some discrepancies exist in the relative change of root-to-shoot under P deficiency, which was be similar in dicot plants but greater in monocot plants compared with P-added soils. Under K deficiency, the data collected did not support a firm conclusion about the root morphology; however, the decrease in root length differed in magnitude between monocot (approx. 10%) and dicot (approx. 2%). It is similar to the study of Samal et al. (2010), who found a contrast in the magnitude of decrease among some crops tested under K deficiency. Therefore, despite the differences in the root architecture among crops and root types, it is highly likely that the fundamental regulators and sensing mechanisms are similar among monocot and dicot species.

To the best of our knowledge, this study considered all retrievable publications investigating root morphology in common crops at the field scale. Publications involved many soil types, weather conditions, management strategies, and genotypes. Furthermore, we showed findings contradictory to pot experiments and revealed the strengths of field-scale studies. Moreover, due to the meta-analysis of individual observations in each publication, we were able to quantify and statistically support the decrease in root length and biomass and the increase in root length per shoot biomass in low N and P environments. Our study had some limitations, though. None of the studies provided data on all the parameters we investigated. However, some studies had the data needed (such as root biomass, shoot biomass and root length) to calculate root-to-shoot data, root length per shoot biomass, and specific root length. We could calculate these ratios for a better comprehension of the deficiency response.

Nevertheless, the most critical limitation was the incompleteness of information about soil properties and nutrient concentration in the soils and crops in many studies. In this regard, our approach was to classify soil as “deficient” when the nutrient was not applied (0 kg ha^-1^), which is not necessarily true depending on the soil nutrient content and the needs of a specific crop. Hence, the unfertilized treatment may or may not lead to nutrient deficiency.

Furthermore, our study did not address relevant interactions that may have an impact on the root morphology in the field, for instance, drought, soil temperature, and soil pH. They remain as open questions for further studies. Additionally, studies did not report about root-soil contact and interaction of roots with the rhizosphere microbiome and potential consequences for plant nutrient acquisition (Wendel et al., 2022) which remains a research gap.

## 5 Conclusions

Our study contributes to the knowledge about root adaptation to nutrient-deficient soils. We detected common mechanisms for how root morphology responds to N, P, and K deficiency, even though roots experience multiple interactions simultaneously in the field. Our main findings point out a decrease in root length and biomass but an increase in root length per shoot biomass and root-to-shoot ratio. These findings are particularly interesting for modelling of root growth and agroecosystem, which requires data about the changes in root traits under different nutrient conditions. Future work must now focus on elucidating interactions of nutrient-driven changes in root architectures with other environmental parameters, such as drought, temperature, the soil microbiome, or soil type. Particular focus could be lain on root nutrient plasticity at field scale, since its assessment with high temporal and spatial resolution is nowadays possible with the emerging non-invasive technologies for root phenotyping.

## Data availability statement

The original contributions presented in the study are included in the article/**Supplementary Material**. Further inquiries can be directed to the corresponding author.

## Author contributions

GL, SS, JP contributed to the conception and design of the study. ASt, GL, SA, SS contributed to the search in scientific databases. GL organized the database, extracted the information, and made calculations and statistical analyses. GL, SS wrote the first draft of the manuscript. JP, WA, GS wrote sections of the manuscript. MA, FE, TG, MG, TK, SR, ASc, MW, PY contributed to improving and correcting the text and figures/tables. All authors contributed to the manuscript revision, read and approved the submitted version.

## Footnotes

1 Plasticity is defined as the reorganization of the root architecture in response to one or several external disturbances that affect and impact the root morphology (Correa et al., 2019).

2 Treatment description: N0 stands for no N fertilizer applied, N(N supply level) stands for the amount of N applied in kg ha^-1^ (e.g. N150: 150 kg N ha^-1^ were applied). This is similar for P and K nutrients (e.g. P0, P44, K0, K30).
