# Supplementary material for: Nutrient deficiency effects on root architecture and root-to-shoot ratio in arable crops
Source: Front Plant Sci. 2023 Jan 4;13:1067498. doi: 10.3389/fpls.2022.1067498 (PMC9846339; doi:10.3389/fpls.2022.1067498)
Supplement: Supplementary file 1 [file DataSheet_1.docx]

Supplementary Material

Supplementary Table S1. Effects of N deficiency and different N application levels on root morphology and root distribution as well as on root biomass.  Treatment description: N0 stands for no N fertilizer applied, N(N supply level) stands for the amount of N applied in kg ha^-1^ (e.g. N150: 150 kg N ha^-1^ were applied). A detailed bibliography of the references is included in the main manuscript.

| **Crop, Site, Soil** | **Treatments and sampling** | **Effects on root morphology and distribution** | **Effect on root biomass and root-to-shoot ratio** | **Reference** |
| --- | --- | --- | --- | --- |
| Crop: Maize. Site: USA, 3yrs.  Soil: silt loam. | Two N rates: N0 and 180 kg N ha^-1^ (N0 and N180). Root sampling:  At ~ 9, 12 and 19 weeks after planting. Root coring (0-0.6m). Root length by counting intersections on a grid. Root diameter using a dissecting microscope. | Lower root length at N0 as compared to N180 in 2 of 3 years. 0N increased the root radius in all three years. | Decreasing of root weight without N fertilization in the 0- to 0.07-m soil layer in one year but slightly increasing (not significant) in two years. Root mass per length decreased with N fertilization. Increasing (significant) of root-to-shoot ratio (0.26-0.40) and rootN-to-shootN ratio (0.61-0.84) at 0N level in all 3 years. | (Anderson, 1987, 1988) |
| Crop: Maize (two genotypes). Site: USA. Soil: silt loam (two field sites). | Two N levels: N0 and 227 kg N ha^-1^ (N0 and N227). Root sampling:  At 31, 47, 75 and 91 days after planting. Coring from row and 35cm from row. Root length was measured by the root intersect method. | Percentage of roots with root hairs was not affected by applied N.  Applied N increased both length and density of root hairs of both genotypes. Root length was higher at N227 for one cultivar but similar for the other as compared to N0. Applied N increased root growth rate at topsoil in early stage (vegetative period). |  | (Barber & Mackay, 1986) |
| Crop: Winter Wheat. Site: England. Soil: silty clay loam | N fertilizer rates: 0, and 154 kg N ha^-1^ (N0, N154).Two levels of water supply: (-1-1, irrigated; - I , drought) | Nitrogen fertilizer increased root lengths by 32 % overall, but drought reduced them by 14%. | Three of the treatments had little effect on total root dry weights, with values in the range of116—134 g/m" at anthesis, but the value for the drought, low N crop was significantly less, at 97 g/m". Low N supply increased the root/shoot weight ratio. | (Barraclough et al., 1989) |
| Crop: Cotton. Site: China. Soil: loam | N rates (0, 120, 240,and 480 kg ha^-1^): N0, N120, N240, N480.Root sampling: Canal drill with an inner diameter of 70 mm, 150cm depth in layers of 15cm.Scanned and analyzed with WinRHIZO | A moderate N fertilization rate (240 kg ha-1) increased root length, root surface area, and root biomass in most soil layers and significantly increased total root growth and total root biomass by more than 36.06% compared to the 0 kg ha^-1^ treatment. In addition, roots in the surface soil layers were more strongly affected by N fertilization than roots distributed in the deeper soil layers. |  | (Chen et al., 2020) |
| Crop: Spring Wheat. Site: UK (two sites)Soil: silt loam, clay loam | N fertilizer rates: 0, 67, and 134 kg N ha^-1^ (N0, N67, And N134).Two sites: Crookston and Morris. Root sampling: Core samples taken with a hydraulic probe (cutting diameter 6.6 cm) centered directly between the rows. | Root length was affected by N fertilization rate for both years and locations. We found that the 67 kg N ha^-1^ rate stimulated root growth within the top 30 cm of the soil profile. The 134 kg N ha^-1^ rate, however, caused either no change or a decline in root length. Genotypes reacted differentially in root length to N fertilization primarily in the surface soil layers at both locations in 1982. Therefore, root growth, although genetically determined, was modified by location differences (i.e., climate, soil types). Since forage production was similar under the 67 and 134 kg N ha^-1^ rates, the decrease in the total root growth observed at the 134 kg N ha^-1^ rate may account for differences observed in water use between N rates |  | (Comfort et al., 1988) |
| Crop: Maize (three genotypes) Site: USA. Soil: Silty clay loam (Typic Argiudol). | Four N rates 0, 60, 120, and 180 kg N ha^-1^ (N0, N60, N120, and N180). Root sampling: At tasseling, roots were sampled with soil cores, (0-0.9m), washing, sieving, drying. | Root dry weight, length and root-to-shoot ratio decreased linearly as N rate increased. Genotypic differences of root dry weight, radius and root-to-shoot ratio were observed. | Increasing of dry weight at 0N (26.3 g per plant) compared with 25, 19 and 20 g per plant at 60, 120, 180 kg N ha-1.Increasing of root-to-shoot ratio at 0N (0.193) as compared to treatments at 60, 120 and 180 kg N ha-1 (0.159, 0.128 and 0.145) | (Eghball & Maranville, 1993) |
| Crop: Maize. Site: China. Soil: medium loam | Four levels of N: 0, 90, 180, and 270 kg N ha^–1^, namely N0, N90, N180, and N270, respectively. Split-plot design: two types of film, (i.e. biodegradable film and conventional polyethylene film).Root sampling: A soil sampler (a 15 × 15 × 10 cm cube) was used for sampling the root system. From 1 to 40 cm in 10cm depth intervals. Scanned and analyzed with WinRHIZO. | The results showed that compared with N0, N application significantly increased root length density (0–20 cm layer), root surface area density (0–10 cm layer), root weight(0–10 cm layer), and root/shoot ratio by 41.6%, 38.8%, 89.8%, and 33.0% on average for biodegradable film and by 40.3%, 33.9%, 61.5%, and 23.3% for polyethylene film, respectively |  | (Fang et al., 2022) |
| Crop: Maize. Site: China, 3 yrs. Soil: three sites: loamy clay, clay loam, and sandy loam | Five fertilizer N rates: 0, 168, 240, 270, and 312 kg N ha^–1^ (N0, N168, N240, N270, And N312) Root sampling: Soil sampling to 0.6m depth at silking. Washing & sieving of roots, scanning and use of image analyzer software. | Soil, and N rate had an effect on maize total root length in the 0- to 60-cm depth. In loamy clay and clay loam soils, there were weak but significant correlations between N supply and total root length. Total root length increased with rising fertilizer N application up to a maximum with 168 to 240 kg N ha–1 and then decreased when N supply further increased. In sandy loam soil, no such a relationship between N supply and total root length was found. | Root biomass at silking was affected by soils and year, but not by N treatment. Root/shoot ratio at silking was not affected by N rate. | (Feng et al., 2016) |
| Crop: Sugar beet. Site: Germany^[[1]](#footnote-1)^. Soil: Silty loam | Three treatments: N0, 160 kg N ha^-1^ including manure (N160), and 80 kg N ha^-1^ (N80) at all applied since decades. Root sampling: At 38, 66, 93 and 155 days after sowing. Excavated with a shovel (topsoil) WinRHIZO | Total root length and root diameter increased with N level in all dates. On date one root diameter was 0.46, 0.44 and 0.41 mm for N0, N80 and N160. Root diameter was lower for N0 as compared to N80 and N160 on sampling dates two and three. | Lower root dry matter in N0 compared with N80 in the first two sampling dates but higher in the last 2 dates. Greatest root dry matter in N160.Highest root-to-shoot ratio in N0 in 3 out of four dates as compared with N80 and N160 (last date: 5 vs 4 vs 3.3 for N0, N80, N160) | (Hadir et al., 2021) |
| Crop: Winter oilseed rape (three cultivars). Site: Belgium.  Soil: Luvisol | Two N levels: N0 and 240 kg N ha^-1^ (N240). Root sampling: At BBCH 15, 18, 19, 55, 65, 75 and 89Tube-rhizotron system. 1m tubes in 45° angle were installed until about 48cm soil depth. Roots in the pictures were redrawn with a digital pen and analyzed in Optimas v6.1. | At BBCH18 (early stage), root length surface density in the deep soil horizon (36-48 cm) was significantly higher in N0 as compared to the N240 treatment There was an important heterogeneity between rhizotron images, but no obvious difference in rooting depth and placement was observed between cultivars across time points and N treatments |  | (Louvieaux et al., 2018) |
| Crop: Maize (two genotypes). Site: USA. Soil: silt loam (two field sites - Raub silt loam and Chalmers silt loam) | Two N levels: N0 and 227 kg N ha^-1^ (N0 and N227). Root sampling:  At 31, 47, 61, 75, 91 and 109 days after planting. Coring from row and 35cm from row. Root length was measured by the root intersect method. | Total root length increased under N227 for one genotype (Pioneer 3732), but It was similar for the other one (B73xMo17). The root growth of B73XMol7 was greater than Pioneer 3732 in the fertilized topsoil (0 to 25 cm). N0 foster the root length in one genotype but not in the other. |  | (Mackay & Barber, 1986) |
| Crop: Winter wheat. Site: Iran. Soil: | Three N treatments: 0 (N0, control), 150 (N150), and 300 (N300) kg N ha^−1^ in 2015/16 and 2016/2017.Two irrigation levels: ordinary furrow irrigation (OFI) and variable alternate furrow irrigation (VAFI). To planting methods: on-ridge planting (ORP) and in-furrow planting (IFP).Root sampling: From the surface layer of the ridges and in the furrows down to 0.8 m soil depth with a hand-driven auger with a length of 1.0 m and 0.05 m diameter (0.2 m depth interval)Washing and root length (m)was determined by the GiaRoots software | Application of nitrogen fertilizer increased the mean RLD compared to that obtained in non-fertilized treatments.N150 showed the higher root density in the upper soil layers and a lower tendency to penetrate to deeper layers than non-fertilized treatment. Generally, both fertilized treatments (N150 and N300) did not indicate a considerable difference between them; however, they were different from non-fertilized treatments | Nitrogen application increased RMD (root mass density) compared with the non-fertilized treatment. In the first year, applying nitrogen fertilizer increased RMD by 32%, whereas it increased RMD by 5% in the second year. | (Mehrabi et al., 2021) |
| Crop: Sorghum. Site: Australia. Soil: Tindall clay loam | Two N levels: 0 (N0) and 100 kg N ha^−1^ (N100).Two hybrids: Texas 610 and Pioneer 846). Root sampling: With a 5-cm auger. Separated in 10cm layers until 150cm.Root length was determined by the line-intersect method |  | There was small difference between treatments in the final total root dry matter levels; all were of the order of 1000 kg ha^-1^, except for Texas 610 in N0 treatment which produced only 600 kg ha^-1^ of roots. | (Myers, 1980) |
| Crop: Sorghum. Site: India. Soil: | Two levels of N fertilizer, 0 (N0) and 100 kg N ha^−1^ (N100) in 1996 rainy season. Two varieties: CSH9 and FSRP. Root sampling: A monolith sampling method - 60 cm depth. Total root lengths were determined with a COMAIR root scanner. | Root length was not much affected by fertilizer-N in hybrid and local variety. The amount of N absorbed by plants of local variety in 100N treatment remained constant after booting | Root dry mass increased with fertilizer-N | (Nakamura et al., 2002) |
| Crop: Maize. Site: USA. Soil: Silt loam | Two rates of N: 0 and 168 kg N ha^-1^ (N0, N168). Two tillage systems, conventional and no-tillage Root sampling: Soil samples taken with an auger (0 to 90cm soil depth) on four time periods. Root length was determined by the line intersection method. | At conventional tillage, treatments with 0N showed an increased in the total root length (from 0-90cm) along the whole growing period. Under no tillage, the difference was only significant at flowering (greater at N0 as compared to N168) |  | (NaNagara et al., 1976) |
| Crop: Sugarcane. Site: Brazil (two field sites). Soil: Typic Kandiudox (TK) Rhodic Eutrudox (RE). | Four N treatments^[[2]](#footnote-2)^: N0 and 120 kg N ha^-1^ (N0 and N120) in the plant cane, 2 N application in the ratoon (N0 and 150 kg N ha^-1^, (N150). Root sampling: At ratoon crop cycle and ~ 125, 145, 211, 225, 274, 288, 349, 369 days after harvesting Root coring up to 0.6m. | At TK site, N fertilization showed slightly greater root density later in the evaluated period | N doses increased the root biomass during the cycle in a Typic Hapludox, although no N effect was found for root growth at the TK site. At the RE site, ratoon N fertilization increased the root-to-shoot ratio at the beginning of the cycle. Later it was similar between the treatments. | (Otto et al., 2014) |
| Crop: Maize. Site: China, 3 yrs. Soil: Silt loam | Four (2007 and 2008) or three (2009) N treatments:  1) 0N as control. 2) N topdressing at and after tasseling 250 kg N ha^-1^ (TDAT), and 3) N topdressing before tasseling (TDBT) 250 kg N ha^-1^, 4) 450 kg N ha^-1^. Root sampling: At 40, 57, 74, 103, 130 and 148 das. Root sampling (auger 0-50 or 60 cm), washing, root length analysis | N deficiency (N0) stimulated root growth in the early growth stage (V8 stage). The total root length peaked before the tasseling, followed by an early decline compared to other treatments with base N fertilizer and N topdressing before tasseling in all three years. The early decline in total root length in N0 was due to early mortality of the early-initiated nodal roots and growth suppression of the later-initiated nodal roots. Although N deficiency (0 N) enhanced embryonic root growth before V12 (the twelfth leaf emerged), it negatively regulated nodal root growth and mortality. In particular, initiation and growth of the 7th whorl of nodal roots of N-deficient plants after the tasseling was almost completely suppressed. |  | (Peng et al., 2012) |
| Crop: Cotton. Site: Georgia-USA (long term experiment)Soil: sandy loam | Three N fertilization rates (0, 60–65, and 120–130 kg N ha1), N0, N60, N120.Three tillage practices [no-till (NT), chisel till (CT), and mouldboard till (MT)]. Root sampling:0- to 120-cm depth from each plot using a hydraulic probe (5 cm diameter. and 120 cm long) - separated into 0- to 15-, 15- to 30-, 30- 60-, 60- to 90-, and 90- to 120-cm lengths |  | Total biomass 15 cm yield, was greater in CT with N60 than in NT with N120.The root biomass did not show significant differences across the treatments, but a significant interaction between tillage and fertilization in cotton in 2000 | (Sainju et al., 2005) |
| Crop: Maize. Site: USA. Soil: silt loam, clay loam | N fertilizer rates: 0, and 157 kg N ha^-1^ (N0, N157).Some genotypes. Root sampling: soil cores60 cm in depth and 5 cm in diameter were taken manually with a sledgehammer using a steel coring tube and plastic liner between two rows. Separated in 10cm layers, scanned, and analyzed with Winrhizon. | Fewer nodal roots were correlated with greater shoot mass under nitrogen stress. Lines with the few, thick nodal root phenotype had significantly greater dry shoot biomass in low nitrogen conditions when compared to lines with the many, thin nodal root phenotype. Better performance in nitrogen stress: a reduced number of developed root nodes (and thus total number of nodal roots emerged in a given period) and increased nodal root cross-sectional area. The phenotype of fewer, thicker nodal roots was associated with deeper root distribution and resulted in greater shoot growth under nitrogen stress. |  | (Schneider et al., 2020) |
| Crop: Potato (two genotypes) Site: Canada. Soil: | Two fertilizer N rates (0 and 150 kg N ha^−1^, (N150)).Root sampling: At 54, 76 and 96 das. Soil cores were taken from 0-15 and 15-30 cm depths. Root washing, photos were taken, analysis with image analyzer software. Drying and weighting of roots. | Root length and RLD did not shown significant differences between the N treatments. | Higher tuber fresh weight in N150 as compared to N0 in third date (both genotypes). Root-to-shoot ratio was increased at 0N as compared to N150. | (Sharifi et al., 2005) |
| Crop: Maize. Site: New Zealand. Soil: Fine sandy loam | Three rates of N: 0, 168, and 672 kg N ha^-1^ (N0, N168 and N672) Wire-netting root containers (20 cm x 51 cm) were inserted to a depth of 91 cm in the field before sowing. One plant was grown per container. Root sampling: Root harvest (0-90cm) at 69, 127, 189 days after planting, drying and weighting. |  | Higher root dry weight at early stage at N0 (14 g per plants) as compared to N168 and N672 (8 and 12 g per plant). Higher root dry weight at grain filling at N0 (26 g per plants) as compared to N168 and N672 (21 g per plant). | (Thom & Watkin, 1978) |
| Crop: Wheat. Site: China. Soil: Loamy (2 sites) and Clay (1site) | Three nitrogen rates depends on the site. Two sites with 0, 180 and 360 kg ha–1, as N0, N180 and N360, respectively. And one site with 4 levels: 0, 180, 240 and 300 kg ha–1 (N0, N180, N240 and N360) Three sites. Two irrigation regimes (no irrigation, W0; and irrigated at the stages of jointing plus booting, 750 m^3^ ha^–1^ each time, W2).Root sampling: digging out soil blocks | At the Kaifeng experimental site, the N180 treatment achieved the highest RWDV values in the 0–20 cm, 20– 40 cm and 40–60 cm soil layers during both growing seasons, while little difference was found among nitrogen treatment groups in the deep soil layers. Under irrigated conditions in Zhengzhou and Wenxian (W2, 2012–2013), the N180 treatment produced the highest RWDV values in the 0–20 cm soil layer, while the N0treatment produced the highest RWDV values in the 60–80 cm and 80–100 cm soil layers. | The effect of nitrogen on RWDV (root weight density) depended on soil water conditions. The application of N180 produced the maximum RWDV under irrigation conditions, but N0 produced the highest value under water stress conditions. Both no-irrigation and N0 treatments significantly increased the R/S, and the combined treatment (W0N0) produced the maximum R/S. The irrigation and nitrogen rate had a significant impact on the RWDV in both Zhengzhou and Wenxian. Under irrigation conditions (W2, 2012–2013), the highest RWDV values in 0–100 cm soil were obtained in the N180 group. Under no-irrigation conditions (W0), however, the N0 treatment produced the highest RWDV values, which gradually decreased in conjunction with the increase in nitrogen application. | (Wang et al., 2014) |
| Crop: Barley. Soil: not provided. Site: England. | Four N treatments: 0, 50, 100 or 150 kg N ha^-1^ (N0, N50, N100 and N150). Root sampling:  At 46, 60, 74, 95, 130 das (days after sowing). Root coring until 0.8m, washed, sieved, cleaned, dried, and weighted. |  | N applications of up to 100 kg ha^-1^ increased the total weight of roots, most of the increase being in the top 15 cm of soil.  Root-to-shoot was higher in treatments N0 and N50 as compared to N100 and N150 (e.g. at date five: 0.09 vs 0.07). Highest root-to-shoot ratio in earliest stage for N0. N applications increased the shoot more than the root weights, thus N applications decreased the root-to-shoot ratio. | (Welbank & Williams, 1968) |
| Crop: Winter wheat. Site: China. Soil: | Four N treatments: 0 (N0, control), 122 (N122), 174 (N174) and 300 (N300) kg N ha^−1^ in 2010/11.Root sampling: Root sampling by extracting them from a soil volume of 30x30cm with a height of 60cm.Washing and scanning of roots, use of image analyzer software | At jointing stage, increasing N from N0 to N174 significantly increased RLD and RSA from 0-30cm and 30-60cm while N122 showed an intermediate but non-significant improvement in RL and RSA. Further increasing N from N174 to N300 did not significantly affect these parameters. At flowering stage, the effects of N on RL and RSA in 0-30 or 0-60 cm soil layers were the same as at jointing stage but less pronounced. At maturity, N supply significantly increased RL and RSA in the 0-30 cm and 0-60 cm soil layers from N0 to N174 (but similar for N174 and N300). | Decrease of root-to-shoot ration with increasing N supply. The N treatments, compared to the N0 rate, resulted in significantly higher root DW (0-60 cm soil depths). At anthesis, root DW at 0–60 cm depths were significantly improved by increasing N from N0 to N122. Root DW was slightly decreased by a further increase of N from N174 to N300. Root DW at 30-60 cm soil depths was not significantly affected by different N treatments at anthesis. At maturity, N supply significantly increased root dry weight (significant for N0 and N300 but quite similar for N174 and N300). | (Xue et al., 2014) |
| Crop: Rice (two hybrid-rice cultivars). Site: China. Soil: clay-loamy soil | Four N: N0, N60, N120, and N180 (kg N ha^-1^) and P levels: P0, P60, P90, and P120 (kg ha^-1^). Root sampling: Soil coring (10cm deep).Washed and sieved. | Both N and P fertilization had a positive impact on root growth up to moderate dose. At early stage (10 days after sowing), P < 90 kg ha^-1^ favored the number of roots in both genotypes. The number of roots is greater in 90 and 120 kg P ha^-1^ later in the growing period in both genotypes. |  | (Yang et al., 2019) |

Supplementary Table S2. Effects of P deficiency and different P application levels on root morphology and root distribution as well as on root biomass. Treatment description: P0 stands for no P fertilizer applied, P(P supply level) stands for the amount of P applied in kg ha^-1^ (e.g. P44: 44 kg P ha^-1^ were applied). A detailed bibliography of the references is included in the main manuscript.

| **Crop, Site, Soil** | **Treatments and sampling** | **Effects on root morphology and distribution** | **Effect on root biomass and root-to-shoot ratio** | **Reference** |
| --- | --- | --- | --- | --- |
| Crop: Soybean (two genotypes) Site: China. Soil: Acidic red soil deficient in P | Two P levels: P0 and 160 kg P ha^–1^ and (P160) Root sampling: At flowering. Camera and image analysis. Scanned and analyzed with WinRHIZO | The two parental genotypes, CN4 and XM6, differed significantly in the seven root morph-architecture traits measured. Root length, root surface area, root volume, root width were significantly higher in the treatment with high P. Specific root length and root depth did not show significant differences between the P treatments. The P-efficient genotype established longer and larger root system with preferring distribution in surface layer and kept more active roots and, therefore, had a better growth performance in field than the P-inefficient genotype. Root volume was higher for high P as compared to low P. |  | (Ao et al., 2010) |
| Crop: Maize. Site: China. Soil: silty loam | Eight P application rates: P0, P12.5, P25, P50, P75, P100, P150 and P300 (kg P ha^-1^). Root sampling: Sampling at flowering | Root length increased with more P supply until reach the critical Olsen-P level indicating a plateau at 8 mg kg. As soil Olsen-P increased from very low levels, specific root length and root-to-shoot ratio declined substantially at first, and then gradually reached a plateau when Olsen-P exceeded 5 mg kg. | In the field experiment, root dry weight also initially increased with increasing soil P supply, peaked when soil Olsen-P was about 2.5 mg kg-1, and then gradually declined to plateau at an Olsen-P level around 10 mg kg-1. | (Deng et al., 2014) |
| Crop: Oilseed rape. Site: China, 2 yrs. Soil: yellow-brown soil (Alfisol). | Two P treatments: 90 kgP2O5 ha^-1^ (sufficient) and30 kg P2O5 ha^-1^ (deficient).Root sampling: Excavated soil cubes (1000 cm3) | Root surface area in 0- 10 cm soil layer and Root length of 2- 5 mm root diameter in 0-10 cm soil layer were significant smaller at deficient P supply. Total root length in 0 - 10 cm soil layer was less with a deficient P supply When plants received a deficient P supply, seed yield had significant positive correlations with coarse root length (diameter 2-3mm), root surface area, and total root length in 0-10 cm soil layer at the flowering stage. The total phosphorus content had significant positive correlations with total root length and root surface area at the flowering stage when plants received a deficient P supply. Plants grown with a deficient P supply had larger total root length ratio and coarse root length ratio in the surface soil (0-10 cm soil layer) and a larger root/shoot ratio at the flowering stage than plants grown with a sufficient P supply. Increased distribution of coarse roots in the surface soil (0-10 cm soil layer) to increase P acquisition at the leaf development and flowering stages | The root_to_shoot ratio was larger when plants were grown with a deficient P supply than when grown with a sufficient P supply | (Duan et al., 2020) |
| Crop: Soybean Site: USA. Soil: fine-silty | Three levels of soil P availability (4, 19 and 32 mg kg^−1^, Mehlich-3 P) and two of levels of water (irrigated and non-irrigated).Root sampling: At 78 das. Root coring (until 36 cm deep) Scanned and analyzed with ROOTEDGE. | P deficiency slowed vegetative development and increased root length density in the surface soil. Increase in root length density with low water availability suggests that water deficit limited P nutrition not by reducing root growth but by decreasing P diffusion in the soil. |  | (Gutierrez-Boem & Thomas, 1998) |
| Crop: Sugar beet. Site: Germany. Soil: Silty loam | Three P levels: P0, 77 kg P ha^-1^ including manure (P77), and 31 kg P ha^-1^ (P31) at all applied since decades.  Root sampling:  At 38, 66, 93 and 155 days after sowing. Excavated with a shovel (topsoil) WinRHIZO | Decreasing total root length under P0 compared with full fertilization, except in the last date. Increasing total root length under P0 compared with no fertilization, except in the second date. | Decreasing of root dry matter in P0 compared with full fertilization, but greater than no fertilization at the beginning and middle stage. Decreasing of root-to-shoot ratio in P0 compared with full fertilization and similar to not fertilized treatment. | (Hadir et al., 2021) |
| Crop: Common beans (two genotypes)  Site: Costa Rica (2 yrs.) | Two sites based on low-phosphorus availability. Phosphorus levels averaged 10 mg kg-1 at Site 1 and 8.3 mg kg-1 at Site 2 (determined by Mehlich-3 extraction). Root sampling: flowering. Soil coring (4com diameter 45 cm depth) | Total root length was greater in low P (about 18%) for one genotype but similar (in low and high P) in the other genotype |  | (Henry, Chaves, et al., 2010) |
| Crop: Common beans (two genotypes) Site: Honduras | Eight sites based on low-phosphorus availability: Low P (3-38 mg P kg-1) and high P (14-252 mg P kg-1), determined by Mehlich-3 extraction. Root sampling: flowering. Soil coring (4 cm diameter 60 cm depth) | The LV4 irrigated site compared to the LV4 drought site: Total root length was not significantly different among genotypes or between single and multilines, but L-88 14 + 43 had on average 80% greater total root length than L-88 14 under drought/high-P, and 44% less total root length than L-88 14 under drought/low-P. In multiline plots, very few roots were found below 45 cm in the high-P treatment whereas root distribution with depth in the low-P treatment was variable. |  | (Henry, Rosas, et al., 2010) |
| Crop: Soybean (several genotypes) Site: China. Soil: acid red soil deficient in P. | Two P levels, 0 and 160 kg P ha^-1^ (P160).Cultivated, semi-wild and wild type genotypes. Root sampling: At flowering. Square block of soil (40cmx40cm) with the plant base at the center was dug to reach the end of tap root. Washed, scanned and analyzed using WinRHIZO pro. | Root architecture was closely related to P efficiency in soybean. The bush cultivated soybean had a shallow root architecture and high P efficiency, the climbing wild soybean had a deep root architecture and low P efficiency, while the root architecture and P efficiency of semi-wild soybean were intermediate between cultivated and wild soybean; Soybean roots became shallower with P addition to the topsoil, indicating that the co-evolutionary relationship between root architecture and P efficiency might be attributed to the long-term effects of topsoil fertilization. Without P addition, most cultivated genotypes had shallow roots and most wild genotypes had deeper roots, and the semi-wild genotypes were mostly intermediate, indicating that the evolutionary pathway of soybean root architecture could be from deep to shallow. However, some semi-wild genotypes became shallow-rooted when P was applied to the topsoil. |  | (Jing et al., 2004) |
| Crop: Maize. Site: Canada. Soil: Deep clay loam soil | Three P fertilization treatments: P0, 17.5 (P17), and 35 kg P ha–1 (P35) Two tillage treatments Root sampling: At silking. Root coring, washing, scanning and analysis with WinRHIZO | In general higher root mass density, root surface density, RLD in high P P1P) but comparing 0.5P and 0P, the values are a bit higher for P0.The root surface density was higher with the 1P than with the 0P and 0.5P application. RLD was 32 % (P> 0.05) higher with the 1P than with the 0P and 0.5P applications. P fertilization showed effects on corn roots by having fewer roots, especially primary roots in 0P and 0.5P. The root length density of primary roots was significantly higher in the high P treatment. | The 35 kg P ha^−1^ rate increased the root biomass by 26 and 41 % compared to the 0 and 17.5 kg P ha^−1^ rates. | (Li et al., 2017) |
| Crop: Common beans. Site: Mozambique. Soil: | Two P levels: 0 and 100 kg P2O5 ha^-1^. Root sampling: Total root length was measured from excavated root crowns of 3 plants per each of four replication at 28 DAP grown in the field | Low P treatment foster the number of adventitious roots. In the topsoil, genotypes with 2 whorls showed greater root length in the treatment with lop P. It was otherwise, in the genotype with 3whorls. In the subsoil the root length was shorter in the low P treatment. Genotypes were also evaluated for root hair length. All genotypes had greater root hair length under low phosphorus availability compared to medium phosphorus availability |  | (Miguel et al., 2013) |
| Crop: Common bean (genotypes with different root phenotype - long-shallow, long-deep, short-shallow, short-deep). Site: Mozambique. Soil: Red loam | The experiment had medium-phosphorus (6 ppm P) and low-phosphorus (19 ppm P). Root sampling:  Root coring (30 cm depth), washed, scanned and analyzed in Winrhizo | Medium P increased the basal root angle, Otherwise low P foster the length of root hairs. |  | (Miguel et al., 2015) |
| Crop: Common bean. Site: Colombia. Soil: | Two P levels: 45 kg ha^-1^ and 7.5 kg ha^-1^. Root sampling: Excavating the plants to reveal as many adventitious and basal roots as possible, and counting the number of adventitious roots emerging from the hypocotyls |  | Lower Biomass and length of adventitious roots under low P level, but greater number of roots and specific root length. | (Ochoa et al., 2006) |
| Crop: Buckwheat, castor, peanut, pigeonpea, sorghum, and soybean. Site: Japan, two sites. Soil: | Two P levels: (P0) had received no P fertilizer since 1979, and the other (P90) received 90 kg P ha^-1^. Root sampling: Through a block dug up along the row at maturity. Root length was measured by the Comair root length scanner | Root length was higher in high P conditions as compared to P0 (exception: pigeonpea). |  | (Otani & Ae, 1996) |
| Crop: Maize. Site: Canada (long-term field). Soil: clay to clay loam | Three P fertilization treatments: P0, 17.5 (P17), and 35 kg P ha^-1^ P35).Two tillage treatments. Root sampling: At the 8 to 10 leaf stage. Root coring, washing, scanning, and analysis using WinRHIZO. | P fertilization had significant effects on total root length density and the percentage of fine and coarse roots. The highest total root length density and percentage of fine roots were observed when 35 kg P ha–1 was added, while the highest percentage of coarse roots was observed when 17.5 kg P ha–1 was added. P fertilization enhanced total root length density, and the percentage of fine roots. |  | (Sheng et al., 2012) |
| Crop: Winter barley. Site: Long-term field. Soil: loamy | Two P levels: 0P and 44 kg P ha^-1^ (44P).Root sampling: At 190, 213, 231, 249 and 268 das. Root coring until 0.3m soil depth. Root production (gross growth) was measured by the ingrowth core method. | The P fertilization resulted in more roots most of the time, however, these differences were significant only at one date and less pronounced than for the shoot development. Higher root length and root weight increment per shoot increment in 0P compared to treatment with P application in all four sampling dates. | Root to shoot ratio was about 60% higher in the 0P treatment over the whole growing period (shoot decrease) 63 and 58% of the total produced roots were already dead at harvest in the 0P and in the treatment with P application. | (Steingrobe et al., 2001) |
| Crop: Common beans. Site: EEUU. Soil: silty loam | Two P levels: low-P (10 ppm mean available P-Mehlich-3) and high-P fields (38 ppm mean available P). Root sampling: Flowering. Soil coring (5.1 cm diameter, 40cm depth), washed, scanned and analyzed with Winrhizo. | Under P stress, reduced genotypes had 32% greater root length density in the top 40 cm of soil than advanced genotypes, while under high P, no genotypic differences were detectable. Root length density in the top 40 cm was positively correlated with total shoot P under P stress, while no relationship was observed under high P. |  | (Strock et al., 2018) |
| Crop: Wheat. Site: China, 2 yrs. Soil: silt fluvo-aquic soil | Six P levels: 0P, 25P, 50P, 100P, 200P, and 400P (kg ha–1) Root sampling: At flowering. Soil volumes of 40 × 20 cm to a total depth of 60 cm were dug out. | RLD increased with P-fertilizer rate at first, peaked at P100, and then declined again (P200 and P400) | Root dry weight increased from P0 to P100 and decreased again towards P200 and P400. Root-to-shoot ration increased from P0 to P400. | (Teng et al., 2013) |
| Crop: Rice (two hybrid-rice cultivars). Site: China. Soil: clay-loamy soil | Four N: N0, N60, N120, and N180 (kg N ha^-1^) and P levels: P0, P60, P90, and P120 (kg ha^-1^). Root sampling: Soil coring (10cm deep).Washed and sieved. | Both N and P fertilization had a positive impact on root growth up to moderate dose. At early stage (10 days after sowing), P < 90 kg ha^-1^ favored the number of roots in both genotypes. The number of roots is greater in 90 and 120 kg P ha^-1^ later in the growing period in both genotypes. |  | (Yang et al., 2019) |
| Crop: Maize. Site: China (long-term field)Soil: loamy and silt (Calcareous alluvial fluvo-aquic) | Two P levels: P0 and 44 kg P ha−1 (P44).Root sampling: At jointing, silking and maturity. A soil volume of 50 cm×20 cm×60 cm was dug out. Scanned and analyzed with WinRHIZO | Increasing length in roots in the soil layers 40 to 60 cm at jointing and in the soil layers 30 to 50 cm at silking without P, especially for the fine roots with the diameter < 0.6 mm. At maturity, however, the root length of the P100 plants in the whole soil profile was longer than that of the P0 plants, regardless of the root diameter. | Lower root weight without P (~2.5g per plant) compared with high N application (~5g per plant) at maturity. | (Zhang et al., 2012) |
| Crop: Maize. Site: USA. Soil: silt loam | Two P treatments: Low P soil averaged 9 +- 0.5 mg P kg–1 and high P soil averaged 120+-13 mg P kg–1.Recombinant inbred lines RILs. Root sampling: Plants were harvested at 32 days after planting to collect lateral root hair length (mm) | Genotypes with long root hairs under low P availability had significantly greater plant growth, P uptake, specific P absorption rates and lower metabolic cost-benefit ratios than short-haired genotypes. Root hair length (mm) was greater in Low P |  | (Zhu et al., 2010) |
| Crop: Common bean (several genotypes). Site: Honduras. Soil: | Four P treatment: i) high P and irrigation, ii) low P and irrigation, iii) high P and no irrigation and, iv) low P and no irrigation. Four recombinant inbred lines (2 with shallow basal root and 2 with deep basal roots) and its parent’s. Root sampling: At 39 to 41 days after planting Root coring until 0.6m.Scanned and analyzed with WinRHIZO. | In the field, shallow-rooted genotypes surpassed deep-rooted genotypes under combined stress conditions (P0 & rain-fed).Total root length density was lower in low P conditions and under rain-fed conditions. Genotypes that allocate roots to surface horizons, particularly basal roots, were better adapted to low P environment. Genotypes that allocate root biomass to deep horizons, particularly to the tap root, are better adapted to terminal drought environments. | No significant differences were found between shallow and deep genotype classes for biomass production. Increasing root-to-shoot ratio under low P conditions. Highest root-to-shoot ratio when P and water were limited. | Ho et al. (2005) |

Supplementary Table S3. Effects of K deficiency and different K application levels on root morphology and root distribution as well as on root biomass. Treatment description: K0 stands for no K fertilizer applied, K(K supply level) stands for the amount of K applied in kg ha^-1^ (e.g. K84: 84 kg K ha^-1^ were applied). A detailed bibliography of the references is included in the main manuscript.

| **Crop, Site, Soil** | **Method** | **Effects on root morphology and distribution** | **Effect on root biomass and root-to-shoot ratio** | **Reference** |
| --- | --- | --- | --- | --- |
| Crop: Soybean. Site: USA, 2yrsSoil: silt loam | 3 K levels^[[3]](#footnote-3)^. Root sampling: At V1, V5, R2, R4 and R6 stage. Root coring, washing, scanning and image analysis | Statistically greater root diameter values in the high-K treatment (2 year mean of 0.274 mm) when compared to low-K (0.252 mm). However, the effect of K treatment on mean root diameter was minor. Calculated root surface area density patterns reflected those of root length density and were not influenced by K treatment. Most K-related differences in above and below ground growth and K uptake rates occurred during early reproductive stages when soil surface water and K availability were high. |  | (Fernández et al., 2009) |
| Crop: Cotton Site: USA, 2yrsSoil: fine sandy loam | Two K treatments: K0 and K84 (kg K ha^-1^).Treatments consisted of K rates broadcast on the surface with and without in-row subsoiling, or deep-placed in the sub­ soil channel. Root sampling: Soil Coring | Root length density decreased from 20 to 80 cm in 0K.In the surface; 20 cm of soil there were no treatment effects on root density in 1990. In 1991, the greatest root densities (0-20 cm) were observed with broadcast K and no subsoiling |  | (Mullins et al., 1994) |
| Crop: Spring Barley. Site: Denmark, 3yrsSoil: coarse-textured meltwater sand | Potassium application at rates of 50, 125 and 200 kg K ha^-1^ (K50, K125, K200) Three irrigation factor: a: Irrigation at 30 mm deficit of soil water (fully irrigated).b: A soil-water deficit of about 50 mm was imposed during early grain filling by shelters. Otherwise as treatment a.c: Irrigated at a soil water deficit of 50 mm if reached. Root sampling: 6.5 cm diameter auger to a depth of 50-80cm in 10cm layer | In 1986 root densities were similar at both K (50K and 200K) levels, while in 1987 the root density in the subsoil layers was significantly (P< 0.04) increased by application of 200 kg K ha-' as compared to the density at 50 kg K ha-1. |  | (Andersen et al., 1992) |
| Crop: maize (C1), sorghum (C2) and millet (C3))Site: Iran, 3yrsSoil: sandy loam soil | Potassium levels: 0 and 200 kg/ha, K0 and K200.Irrigation levels: with estimate leaf relative water content (RWC) by > 95% (non-drought stress condition (S1)) and irrigation under RWC = 60-70% (drought stress condition (S2)).Root sampling: Auger (Height = 100 cm and Radius = 15 cm). 24hrs after irrigation. | Higher root penetration under K Application. Root length was 111.04 cm under K0 while K200 showed 126.12 cm .K application contributed plants to approach longer root length as well through the condition of drought stress |  | (Valadabadi & Farahani, 2009) |
| Crop: maize. Site: China. Soil: sandy | Two levels of Potassium: 0 and 150kg of sulfuric acid potassium per ha .Root sampling: Roots for morphology measurement were sampled at seedling, shooting, booting, and tasseling and flowering stages. Measured with WinRhizo. | Under low potassium (–K) treatment, the total root length of 90-21-3 (tolerance to K deficiency) were decreased before booting stage, whereas it was increased 274 and 347 cm at shooting and tasseling and flowering stages, respectively. By contrast, the total root length of D937 (sensitive to K deficiency) were slightly decreased compared with high potassium (+K) treatment during the whole growing period. Under –K treatment, the total root lengths of 90-21-3 were significantly longer than that of D937 during the later growing period. It was noteworthy that an abundant of lateral roots in 90-21-3 were developed at tasseling and flowering stage under –K treatment, which were significantly more than those of D937 |  | (Zhao et al., 2016) |
| Crop: Sugar beet. Site: Germany. Soil: Silty loam | Three treatments: N0, 182 kg K ha-1 including manure (K182), and 116 kg K ha^-1^ (N116) at all applied since decades. Root sampling: At 38, 66, 93 and 155 days after sowing. Excavated with a shovel (topsoil) WinRHIZO | Lower total root length in treatment NP_Ca compared with NPKCa+manure and NPKCa, except in the last date. Higher total root length in NP_Ca compared with no fertilization, except in the first sampling date. Lower average root diameter in NP_Ca compared to full fertilization (NPKCa+manure & NPKCa) and no fertilization treatment. | Lower root dry matter (g plant-1) in NP_Ca compared to full fertilization, but similar to no fertilization. Greatest root dry matter in NPKCa + manure. Lower root-to-shoot ratio in NP_Ca compared with full fertilization (NPKCa+manure & NPKCa) and not fertilized treatment | (Hadir et al., 2021) |


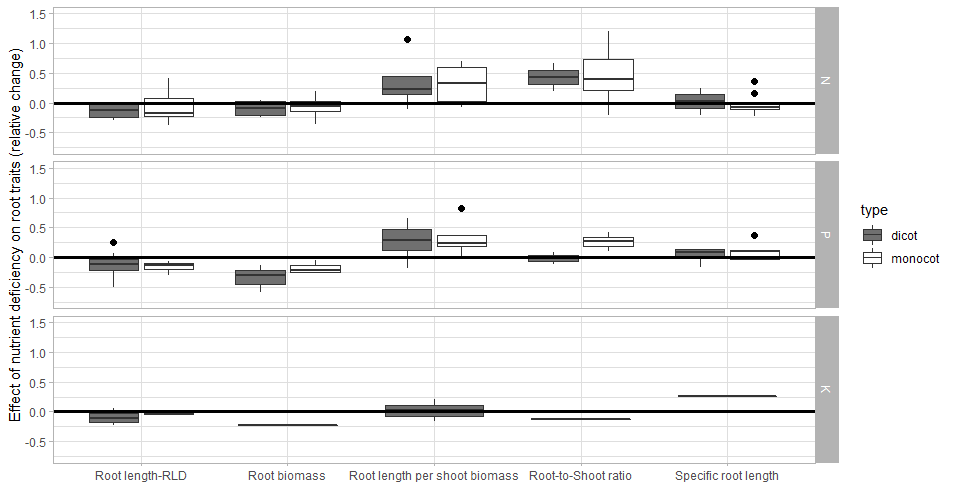


Figure S1. The relative change of the root traits per type of root (monocot-dicot) under deficiency [(X0-X1)/X1] where X0 is the value in the treatment without any addition of the nutrient and X1 is the value of the treatment with the nutrient application

1. Long-term field experiment since 1904. Silty loam (topsoil) and (silty) clay loam (subsoil). [↑](#footnote-ref-1)
2. Four N treatments: 0 and 120 kg N ha^−1^ at sowing, and after the first harvest (4 months) 0 and 150 kg ha^−1^ N (treatments: 0 -0, 0 - 150, 120 - 0, and 120 - 150). Root sampling: Coring near the place of biomass collection, 0.3m and 0.6m apart from that. [↑](#footnote-ref-2)
3. Cumulative K fertilizer applied to individual treatment plots prior to 2002 ranged from 0 to 900 kg K ha−1 creating a broad range of soil K levels. Following 2002, no additional fertilizer K was added to the plots. The ranges in values of soil extractable-K used to assign each fertility treatment were as follows: 54 to 73 mg kg−1 in surface (0 to 10 cm soil depth increment) and 46 to 57 mg kg^−1^ in subsurface (10 to 20 cm soil depth increment) for low fertility; 107 to 164 mg kg−1 in surface and 60 to 70 mg kg−1 in subsurface for medium fertility; and 266 to 316 mg kg^−1^ in surface and 70 to 104 mg kg−1 in subsurface for high fertility [↑](#footnote-ref-3)
